# Atomic-Scale Time-Resolved Imaging of Krypton Dimers, Chains and Transition to a One-Dimensional Gas

**DOI:** 10.1021/acsnano.3c07853

**Published:** 2024-01-22

**Authors:** Ian Cardillo-Zallo, Johannes Biskupek, Sally Bloodworth, Elizabeth S. Marsden, Michael W. Fay, Quentin M. Ramasse, Graham A. Rance, Craig T. Stoppiello, William J. Cull, Benjamin L. Weare, Richard J. Whitby, Ute Kaiser, Paul D. Brown, Andrei N. Khlobystov

**Affiliations:** † School of Chemistry, University of Nottingham, Nottingham NG7 2RD, United Kingdom; ‡ Electron Microscopy Group of Materials Science, Central Facility for Electron Microscopy, Ulm University, Ulm 89081, Germany; § School of Chemistry, University of Southampton, Southampton SO17 1BJ, United Kingdom; ∥ Nanoscale and Microscale Research Centre, University of Nottingham, Nottingham NG7 2QL, United Kingdom; ⊥ SuperSTEM Laboratory, SciTech Daresbury Campus, Daresbury WA4 4AD, United Kingdom; # School of Chemical and Process Engineering and School of Physics and Astronomy, University of Leeds, Leeds LS2 9JT, United Kingdom; ∇ Centre for Microscopy and Microanalysis, The University of Queensland, Brisbane, Queensland 4072, Australia; ○ Department of Mechanical, Materials & Manufacturing Engineering, University of Nottingham, Nottingham NG7 2RD, United Kingdom

**Keywords:** endohedral fullerenes, transmission electron microscopy, time-resolved imaging, carbon nanotubes, noble gases, single-atom dynamics, one-dimensional gas

## Abstract

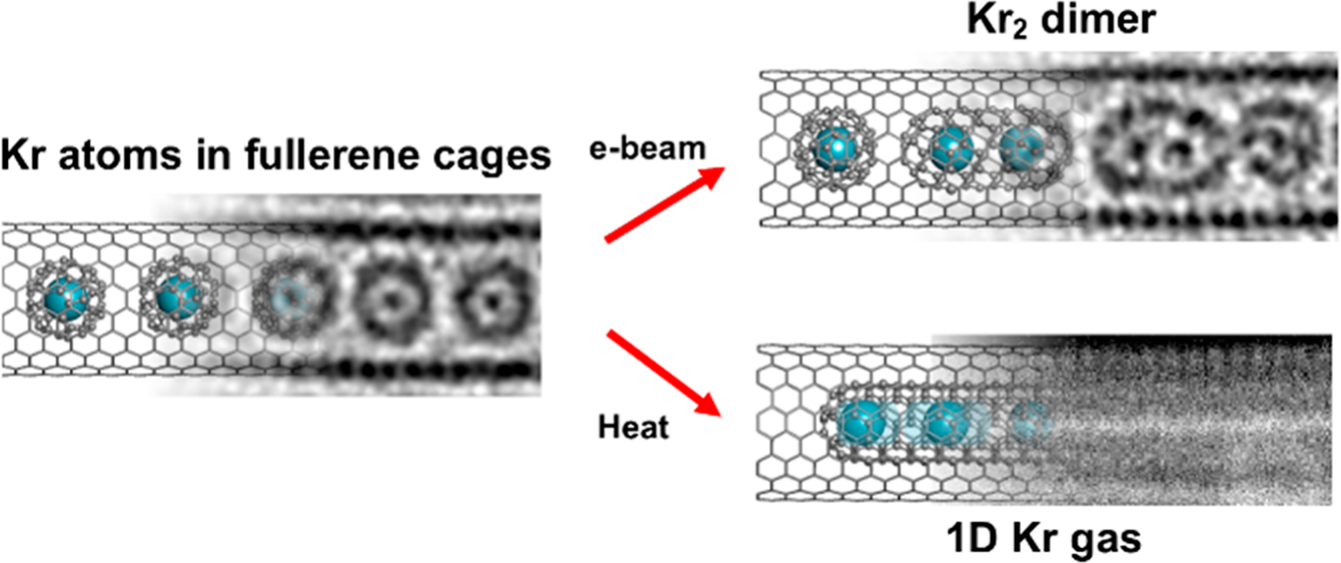

Single-atom dynamics of noble-gas elements have been investigated using time-resolved transmission electron microscopy (TEM), with direct observation providing for a deeper understanding of chemical bonding, reactivity, and states of matter at the nanoscale. We report on a nanoscale system consisting of endohedral fullerenes encapsulated within single-walled carbon nanotubes ((Kr@C_60_)@SWCNT), capable of the delivery and release of krypton atoms on-demand, via coalescence of host fullerene cages under the action of the electron beam (*in situ*) or heat (*ex situ*). The state and dynamics of Kr atoms were investigated by energy dispersive X-ray spectroscopy (EDS), electron energy loss spectroscopy (EELS), and X-ray photoelectron spectroscopy (XPS). Kr atom positions were measured precisely using aberration-corrected high-resolution TEM (AC-HRTEM), aberration-corrected scanning TEM (AC-STEM), and single-atom spectroscopic imaging (STEM-EELS). The electron beam drove the formation of 2Kr@C_120_ capsules, in which van der Waals Kr_2_ and transient covalent [Kr_2_]^+^ bonding states were identified. Thermal coalescence led to the formation of longer coalesced nested nanotubes containing more loosely bound Kr_*n*_ chains (*n* = 3–6). In some instances, delocalization of Kr atomic positions was confirmed by STEM analysis as the transition to a one-dimensional (1D) gas, as Kr atoms were constrained to only one degree of translational freedom within long, well-annealed, nested nanotubes. Such nested nanotube structures were investigated by Raman spectroscopy. This material represents a highly compressed and dimensionally constrained 1D gas stable under ambient conditions. Direct atomic-scale imaging has revealed elusive bonding states and a previously unseen 1D gaseous state of matter of this noble gas element, demonstrating TEM to be a powerful tool in the discovery of chemistry at the single-atom level.

Microscopy is an important analytical tool in chemistry, as direct imaging of atoms and molecules can provide for the discovery of chemical processes at the nanoscale. Transmission electron microscopy (TEM) enables the detection of individual atom positions, with electrons acting simultaneously as an imaging probe and an energy source to drive chemical transformations *in situ*.^1^ The combination of energy selection with high spatial and temporal resolution can facilitate the direct study of chemical processes at the atomic level, in direct space and real time. Accordingly, TEM may be used to record fundamental mechanisms, e.g., bond breaking and formation, creating opportunities to elucidate chemical processes at the single-atom level, provided a suitable encapsulating system is utilized. Investigation of atoms and molecules by TEM inside carbon nanotubes, the world’s smallest test tubes, is particularly informative, as electron beam damage to the sample system is minimized.^2,3^

Our approach to study the interactions between single atoms arises from endohedral buckminsterfullerenes, molecular carbon cages containing individual guest atoms or molecules within their internal cavities (denoted X@C_60_), in turn encapsulated within single-walled carbon nanotubes (SWCNT) forming so-called “peapod” nanostructures, (X@C_60_)@SWCNT. Such structures adopt a linear arrangement of closely spaced carbon cages, each containing a single heteroatom (Figure 1). Upon coalescence of the fullerene cages, driven by the electron beam^4^ or heat,^5^ larger molecular capsules are formed, containing combinations of individual guest atoms or molecules, the interactions between which can be studied in isolation free from the effects of external stimuli (Figure 1). The electron beam directs the local fusion of endohedral fullerenes to initially form 2X@C_120_ capsules, allowing the study of X···X interactions, while thermal treatment more readily enables the study of longer encapsulated atomic chains, *n*X@C_60*n*_ (*n* > 2), and also atoms with one degree of translational freedom, transitioning to a 1D gas state.

**Figure 1:**
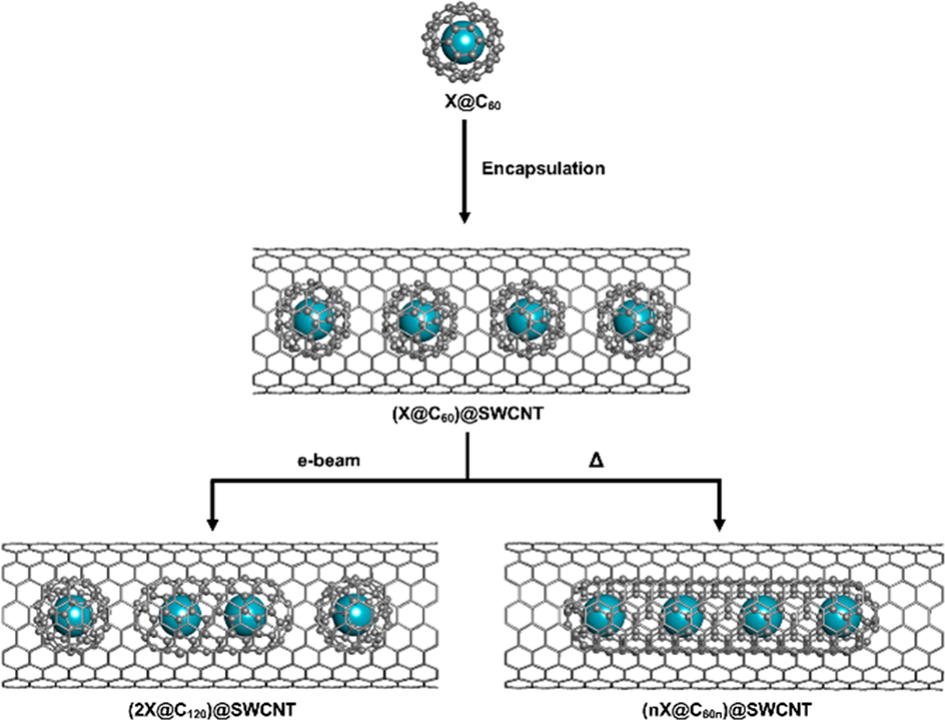
Endohedral fullerenes are introduced into the internal cavity of a SWCNT via sublimation, forming a one-dimensional, linear “peapod” chain. Highly energetic electron beam irradiation, or heat, promotes the coalescence of adjacent endohedral fullerene molecules to form molecular capsules containing short chains of atom X, providing for the study of bonding-level interactions in isolation.

This approach to create a nanoscale system for the delivery, release, and direct study of the bonding states of noble gas atoms builds upon previous work charting the positions of lanthanide metal atoms, following electron-beam-induced coalescence of Dy@C_82_,^6^ La@C_82_,^7,8^ Pr_2_@C_72_,^9^ Sm@C_82_,^10^ and Gd_(1–2)_@C_92_ and Tb_2_@C_92_ inside CNT,^11^ raising the potential use of endohedral fullerenes to control the position and release of single atoms. The effect of encapsulated metal atoms within fullerenes on their coalescence rate have been studied by Koshino *et al.* for La@C_82_ and Er@C_82_.^12^ Further, both the rate and mechanism of fullerene coalescence for the molecular endohedral species HF@C_60_ and H_2_O@C_60_ have been reported by Biskupek *et al.*(13)

Historically, beam damage has been considered a drawback in TEM, obstructing the acquisition of meaningful native state structural information.^14^ However, the controlled utilization of energy transfer in TEM to coalesce endohedral fullerenes builds upon the ChemTEM and related SMART-TEM methodologies, whereby energy transfer to trigger chemical reactions and imaging are combined for the time-resolved study of chemical processes at the molecular level *in situ*.^1,15^ Systems studied by this technique include polyoxometalates (POMs),^16^ perchlorocoronene (PCC),^17^ metal halide nanoclusters,^18^ C_60_,^19^ and diatomic metal clusters.^20^ This level of control afforded over individual atom interactions constitutes a significant step toward the “atom-forge” concept, whereby the electron beam can be used to control the positions of individual atoms and create complex objects.^21^

In this work, TEM is applied to investigate the atomic-scale dynamics and bonding of the noble gas krypton. Previous investigations of krypton atoms by microscopy involved entrapment of Kr by ion implantation in bilayer graphene.^22−24^ Additionally, Kr gas sealed in several-nanometer-wide SWCNT was studied by HRTEM; however, no atomic contrast was observed due to the low filling density and high Kr atom mobility.^25^ In this work, high-purity endohedral fullerene Kr@C_60_ prepared via molecular surgery^26^ serves as a starting point allowing the effective filling of SWCNT cavities with carbon cages, each containing one atom of Kr. In contrast to previously studied lanthanide metal endohedral fullerenes, Kr only very weakly interacts with the carbon atoms of the host cage and so remains encapsulated even after extreme heat treatment at 1200 °C.

It is recognized that any gaseous atom–atom interaction is difficult to study by any means. However, by mediating the fusion of two (or more) Kr-containing fullerene cages, Kr atoms can be released on demand in a controlled manner, facilitating the TEM investigation of atom–atom interactions and bonding in real time and direct space. Electron-beam-induced coalescence to form 2Kr@C_120_ species provides for the investigation of Kr–Kr interactions in isolation, demonstrating the formation of van der Waals dimers, and also the transient existence of covalent diatomic molecular [Kr_2_]^+^. Coalescence of several carbon cages driven by heat facilitates the release of the endohedral atoms into longer containers, where Kr *n*-atom chains behave as a one-dimensional gas. Our work allows the direct observation of bonding states of Kr atoms, thereby expanding the spatiotemporally continuous imaging of atomic scale dynamics to noble gas elements.

## Results

### Characterization of (Kr@C_60_)@SWCNT

**Scheme 1:**
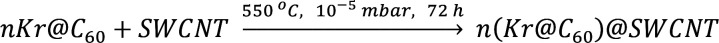
Filling of Kr@C_60_ into Open-Ended SWCNT to Form (Kr@C_60_)@SWCNT

This work aimed to investigate interatomic dynamics and bonding; hence, it was important to deliver target atoms into SWCNT as a stable compound with well-defined composition and structure. This was achieved by molecular surgery, whereby an orifice in the C_60_ cage is opened, followed by Kr atom encapsulation and subsequent cage resealing, via a series of chemical transformations, yielding endohedral Kr@C_60_ with >99% purity.^26,27^ Kr@C_60_ is the first stable compound of krypton, such that it can be heated to 550 °C without the loss of Kr and hence efficiently sublimed into SWCNT of average diameter ∼1.4 nm, thus delivering noble gas into the nanoscale cavities, forming (Kr@C_60_)@SWCNT as shown in Scheme 1.

**Figure 2:**
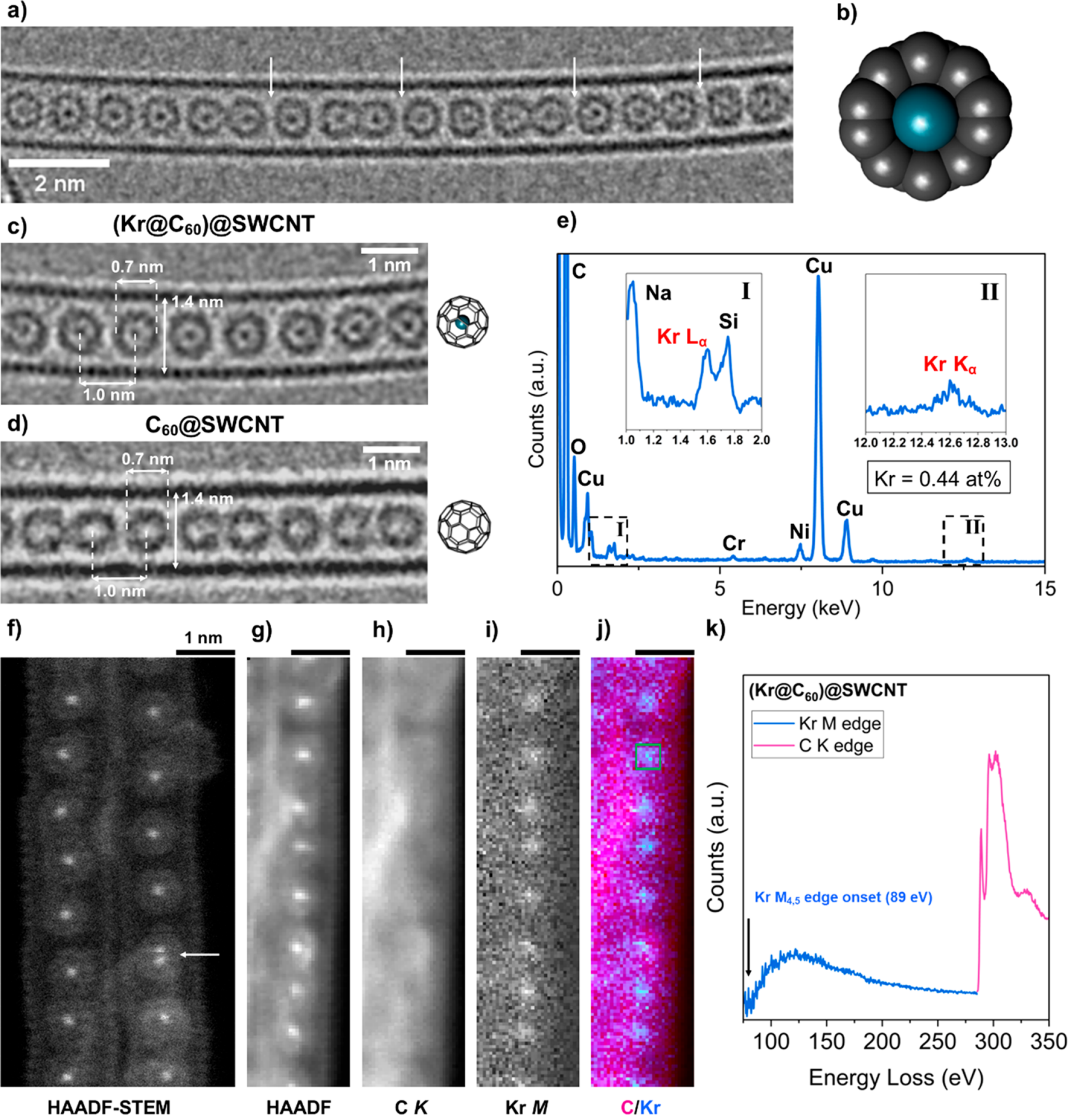
(a–e) AC-HRTEM data for (Kr@C_60_)@SWCNT, recorded at an acceleration voltage of 80 kV, and (f–k) HAADF-STEM data for (Kr@C_60_)@SWCNT, recorded at 60 kV. (a) A freestanding SWCNT fully filled with Kr@C_60_, illustrating the onset of coalescence between adjacent fullerene pairs (arrowed). Additional HRTEM images are shown in Figures S1 and S2. (b) Schematic cross-section of Kr@C_60_ depicting the van der Waals diameters of C and Kr (Avogadro software). (c, d) Representative TEM images of pristine (Kr@C_60_)@SWCNT and C_60_@SWCNT and stick models of Kr@C_60_ and C_60_, respectively. (e) EDS data for (Kr@C_60_)@SWCNT (enlarged **I**, Kr Lα (1.6 keV); **II**, Kr Kα (12.6 keV) peaks inset). Additional recorded signals were attributed to residual Ni catalyst from SWCNT synthesis, O and Cu from the support film and TEM grid, Na and Si from the glass ampule used during SWCNT filling, and Cr from steel in the TEM column. Additional STEM-EDS mapping is shown in Figure S4. (f, g) HAADF-STEM images of bundles of (Kr@C_60_)@SWCNT peapods (adjusted γ = 0.55) where (g) was acquired simultaneously with the EEL signal. Molecular motion during a scan results in a double point (arrowed). (h, i) EELS maps of the C K-edge (283–394 eV) (h) and the Kr M-edge (89–200 eV) (j) False-colored composite map showing the EELS signal from C (magenta) and Kr (blue). The map was created by integrating the intensity of the C and Kr edges averaged at each pixel of the image spectrum. (k) EEL spectrum following background subtraction showing the Kr M-edge and C K-edge averaged over the pixels of the green box in (j). The EEL spectrum is shown without background subtraction in Figure S5.

Figure 2a is an aberration-corrected high-resolution TEM (AC-HRTEM) image, recorded at 80 kV, showing the structure of (Kr@C_60_)@SWCNT. Individual endohedral Kr atoms exhibit strong contrast at the center of each C_60_ molecule. In the HRTEM interference phase contrast imaging mode under these relatively simple conditions (with mostly single atoms), the strength of scattering is approximately proportional to , where *Z* is the atomic number of the scattering atom.^28^ High Kr@C_60_ purity is confirmed by TEM images, where nearly all the C_60_ cages were filled. Each Kr atom lies close to the geometric center of its host C_60_ cage. The close match in size between the van der Waals diameter of Kr (0.404 nm)^29^ and the internal cavity of C_60_ (∼0.4 nm) (Figure 2b) results in symmetrical electronic repulsion, forcing the Kr nucleus to the center, in turn leading to enhanced Kr atom definition due to dampened atomic vibration, reducing motion blur within the cage. Further, Figure 2c,d indicates no difference in the diameter of C_60_ cages (∼0.7 nm), or spacing between adjacent cages (∼1.0 nm center-to-center), when comparing (Kr@C_60_)@SWCNT and C_60_@SWCNT. This reinforces the conclusion that carbon cages in Kr@C_60_ and empty C_60_ are effectively indistinguishable, supported by the small difference in ^13^C NMR chemical shift previously determined by Hoffman *et al.*(26)Figure 2e presents energy dispersive X-ray spectroscopy (EDS) data for a bundle of (Kr@C_60_)@SWCNT suspended over a hole in the TEM grid support film, confirming the presence of Kr at an abundance relative to carbon of ∼0.44 atomic % (at%). This is consistent with the anticipated abundance of Kr:C of 1:240 (i.e., ∼0.42 at%) for a single 1.4 nm diameter SWCNT fully filled with Kr@C_60_ (Figure S3). This combined evidence confirms that Kr@C_60_ is pure and that it encapsulates into SWCNT as effectively as empty C_60_, as previously observed for other (H_2_O@C_60_)@SWCNT and (HF@C_60_)@SWCNT endohedral peapod structures.^13^

Kr atomic position and identity were also confirmed by aberration-corrected high-angle annual dark field scanning transmission electron microscopy (HAADF-STEM) and electron energy loss spectroscopy (EELS). Figure 2f,g presents HAADF-STEM images of (Kr@C_60_)@SWCNT recorded at 60 kV to minimize beam-induced damage, hence aiding native state preservation.

Here, scattering is dominated by large-angle incoherently scattered electrons, where the image intensity is approximately proportional to *Z*^2^, hence Kr atoms appear much brighter relative to C atoms of the host C_60_ cages and SWCNT, enabling the further confirmation of the position and degree of filling of Kr within C_60_ from AC-HRTEM imaging.

In addition, Figure 2h–j present STEM-EELS mapping of the area of (Kr@C_60_)@SWCNT displayed in Figure 2g, showing (Figure 2h) C K-edge and (Figure 2i) Kr M-edge regions, respectively, and (Figure 2j) a false color composite C/Kr map, illustrating unambiguously the presence of individual Kr atoms within each C_60_ cage in this area. Figure 2k shows the corresponding EEL spectrum (integrated over the area highlighted by the green box in Figure 2j), with the Kr M_4,5_-edge onset at 89 eV. Molecular motion during a scan or spectrum image acquisition can limit the level of information, especially if the gaps between molecules are larger than the van der Waals spacing of 0.3 nm. However, this is minimal in densely filled nanotubes, such as presented in Figure 2, and these data combined therefore confirm the high abundance of individual Kr atoms within carbon nanotubes, periodically separated by a distance corresponding to the van der Waals diameter of the C_60_ cage.

Accordingly, this (Kr@C_60_)@SWCNT system acted as a platform for dynamic investigations in AC-HRTEM time-resolved imaging (due to a faster image acquisition rate than in STEM mode), while noting that the electron fluence required for image formation initiated the onset of localized chemical transformations (Figure 2a, arrowed).

### *Ex Situ* Release of Kr Atoms: Thermal Coalescence of Kr@C_60_

**Scheme 2:**
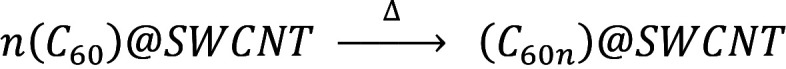
Thermal Coalescence of C_60_ within SWCNT to Form C_60*n*_ Nested Nanotubes

Fullerene cages in C_60_@SWCNT are known to undergo thermal coalescence and annealing into longer cages at elevated temperature (800–1200 °C) to form long, straight nested nanotubes, with the degree of polymerization (fraction of fused molecules) dependent on both the time and temperature of the reaction (Scheme 2).^30^

**Scheme 3:**
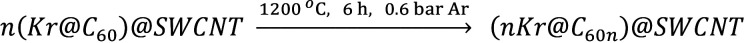
Thermal Coalescence of Kr@C_60_ within SWCNT to Form *n*Kr@C_60*n*_ Nested Nanotubes

This thermal transformation was utilized to coalesce carbon cages in (Kr@C_60_)@SWCNT (Scheme 3), providing for the controlled release of Kr atom chains in the resultant nested nanotubes (Figure 1).

**Figure 3:**
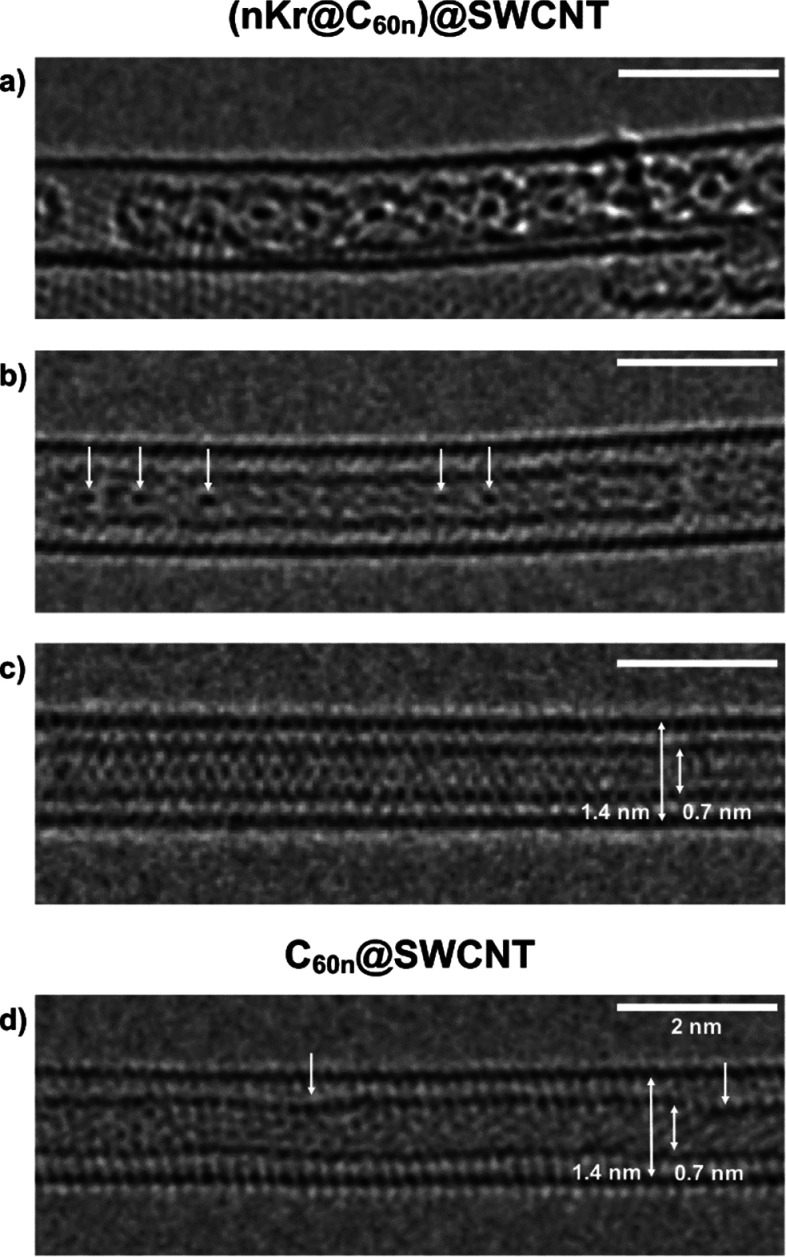
(a–d) 80 kV AC-HRTEM images of (a–c) (*n*Kr@C_60*n*_)@SWCNT and (d) (C_60*n*_)@SWCNT, formed by coalescence of Kr@C_60_ inside SWCNT at 1200 °C. (a–c) Representative areas of (*n*Kr@C_60n_)@SWCNT illustrating different extents of thermal coalescence and annealing of a nested nanotube, from highly corrugated with high barriers for Kr motion (a), to smoother where Kr atoms are partly delocalized (b), to near perfect where Kr can translate freely along the nanotube (c). Figure S6 shows a representative HRTEM survey image highlighting the range of thermally coalesced nested nanotubes. Analogous thermal coalescence of C_60_ in SWCNT forms C_60n_@SWCNT (d), with a similar annealed nested nanotube structure, with arrows denoting localized defects.

Figure 3a–d compares AC-HRTEM images of sections of coalesced endohedral Kr@C_60_ (a–c) and C_60_ (d) molecules, formed during *ex situ* heat treatment at 1200 °C for 6 h under an argon atmosphere.

The application of this heat treatment protocol to (Kr@C_60_)@SWCNT provided for the release of several Kr atoms into a nanoscale, 1D container in the form of a capped nested carbon nanotube. In particular, the representative AC-HRTEM images shown in Figure 3a–c, recorded from the same (*n*Kr@C_60n_)@SWCNT sample, are illustrative of the different extents of annealing of the fullerene cages. Retention of encapsulated Kr atoms was confirmed by EDS (Figure 5b). In bright-field TEM, Kr atom visibility was found to be dependent on the extent of fullerene fusion and the degree of annealing of nested nanotube walls, as defects created mechanical pinning points, preventing Kr atom translation. Figure 3a shows an example section of many coalesced Kr@C_60_ molecules, following partial annealing, with the formation of a “corrugated” nested nanotube with sequential bottlenecks between which Kr atoms were constricted and clearly distinct. Figure 3b shows a section of more fully annealed (*n*Kr@C_60n_)@SWCNT, closer in structure to an extended nanotube with straight walls, which allowed for freer translation of the guest Kr atoms, and hence only some remained visible on the time scale of data acquisition (arrowed).

For completeness, Figure 3c illustrates a fully annealed section of (*n*Kr@C_60*n*_)@SWCNT, with near-perfect straight parallel walls and diameter 0.7 nm, commensurate with the internal cavity of the host SWCNT and diameter of the starting C_60_ cages. In such instances, Kr atoms were highly mobile and no longer visible on the time scale of imaging (0.5 s exposure per frame). Considering that the van der Waals diameters of Kr and C atoms are ∼0.4 and ∼0.3 nm, respectively,^29^ only one translational degree of freedom is available to the noble gas in (*n*Kr@C_60*n*_)@SWCNT with a low energy barrier to translation, suggesting the transition to a one-dimensional gaseous state of Kr atoms. Figure 3d shows a thermally annealed section of C_60*n*_@SWCNT, confirming the development of a well-defined, smooth, hemispherically capped nested nanotube of length >30 nm and diameter ∼0.7 nm, as C_60_ transformed to a thermodynamically more stable carbon lattice with a lower pyramidalization angle, while noting a few residual localized defects (arrowed).

**Figure 4:**
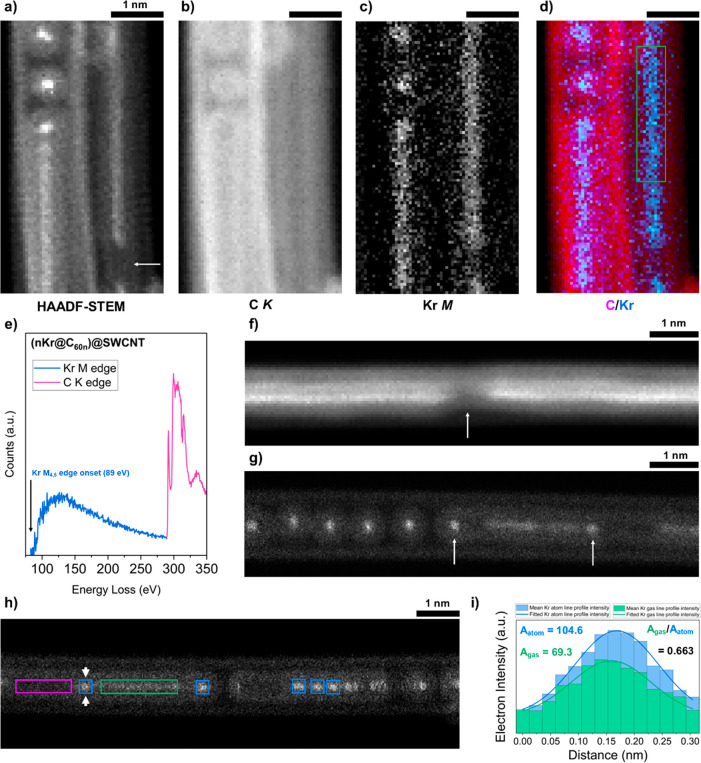
60 kV HAADF-STEM data of (*n*Kr@C_60*n*_)@SWCNT formed by coalescence of Kr@C_60_ inside SWCNT at 1200 °C. (a) HAADF-STEM image of a bundle of (*n*Kr@C_60*n*_)@SWCNT (adjusted γ = 0.40), with bright lines in the center of the nested nanotubes corresponding to highly mobile Kr atoms. (b–d) EELS maps acquired simultaneously with HAADF image (a), showing the C K-edge (283–394 eV) (b) and the Kr M-edge (89–200 eV) (c). (d) False-colored composite map showing the EELS signal from C (magenta) and Kr (blue). The map was created by integrating the intensity of the C and Kr edges averaged at each pixel of the image spectrum. (e) EEL spectrum following background subtraction showing the Kr M-edge and C K-edge averaged over the pixels of the green box in (d). The EEL spectrum is shown without background subtraction in Figure S7. (f, g) HAADF-STEM images of an area of (*n*Kr@C_60*n*_)@SWCNT with a central defect (arrowed), highlighting how the 1D Kr gas cannot transit through such bottlenecks (j), and a short area of 1D Kr gas bounded on either side by stationary pinned Kr atoms (arrowed) (g). (h, i) Calculation of the relative intensity of gaseous Kr atoms (green box) versus stationary Kr atoms (blue boxes) in HAADF-STEM image (h). (i) Histograms of mean per atom integrated intensity in (h), with fitted Gaussian curves. The relative intensity of gas atom intensity to stationary atom intensity is ∼0.66, close to the expected average occupancy of Kr atoms of 2/3 within nested nanotubes.

Highly mobile Kr atoms no longer visible in bright-field TEM were studied further by HAADF-STEM and STEM-EELS mapping. Figure 4a presents a HAADF-STEM image of a bundle of (*n*Kr@C_60*n*_)@SWCNT recorded at 60 kV, where mobile Kr atoms are identified as a continuous line of increased brightness in the center of the nested nanotube in the right-hand and bottom half of the left-hand CNT. Highly mobile Kr atom visibility in HAADF-STEM was attributed to the combination of single-atom brightness approximately proportional to *Z*^2^ and fast scan rate (μs dwell time per pixel). Figure 4b–d presents STEM-EELS mapping of the same area as in Figure 4a, showing (b) C K-edge and (c) Kr M-edge regions, respectively, and (d) a false-color composite C/Kr map, confirming the retention and identity of mobile Kr atoms free to translate post thermal coalescence of fullerene cages, behaving as a 1D gas. Figure 4e shows the corresponding EEL spectrum (integrated over the area in the green box in Figure 4e), with Kr M_4,5_-edge onset at 89 eV. Figure 4f illustrates a long, well-annealed section of a nested nanotube (structurally similar to Figure 3c), where mobile Kr atoms fill almost the entire length, but a defect forms a bottleneck past which Kr cannot transit (arrowed), as the van der Waals diameter of Kr fills entirely the 0.7 nm diameter of the annealed nanotube. Figure 4g shows an area with several intact Kr@C_60_ molecules on the left-hand, and a more defective nested nanotube on the right-hand (structurally similar to Figure 3b). Here, two Kr atoms are pinned in place by defects in the nested nanotube (arrowed), between which mobile Kr atoms behave as a short section of 1D gas. Figure 4h,i presents quantitative measurement of the relative intensity between mobile and stationary Kr atoms in the HAADF-STEM image (h). Figure 4i shows the per-atom histograms averaged over five stationary Kr atoms (blue boxes) and four mobile Kr atoms (green box), fitted Gaussian curves of which yield a relative brightness of 0.66, which closely matches the average expected occupancy of Kr atoms when freely translating within nested nanotubes of 2/3 from a consideration of atomic diameter (Figure S8). As such, STEM analysis has shown Kr atoms can transition to a state with one degree of translational freedom and occupy completely the available volume of the nested nanotube, hence confirming the transition of single Kr atoms to a 1D gaseous state following thermal coalescence to form annealed nested nanotubes.

**Figure 5:**
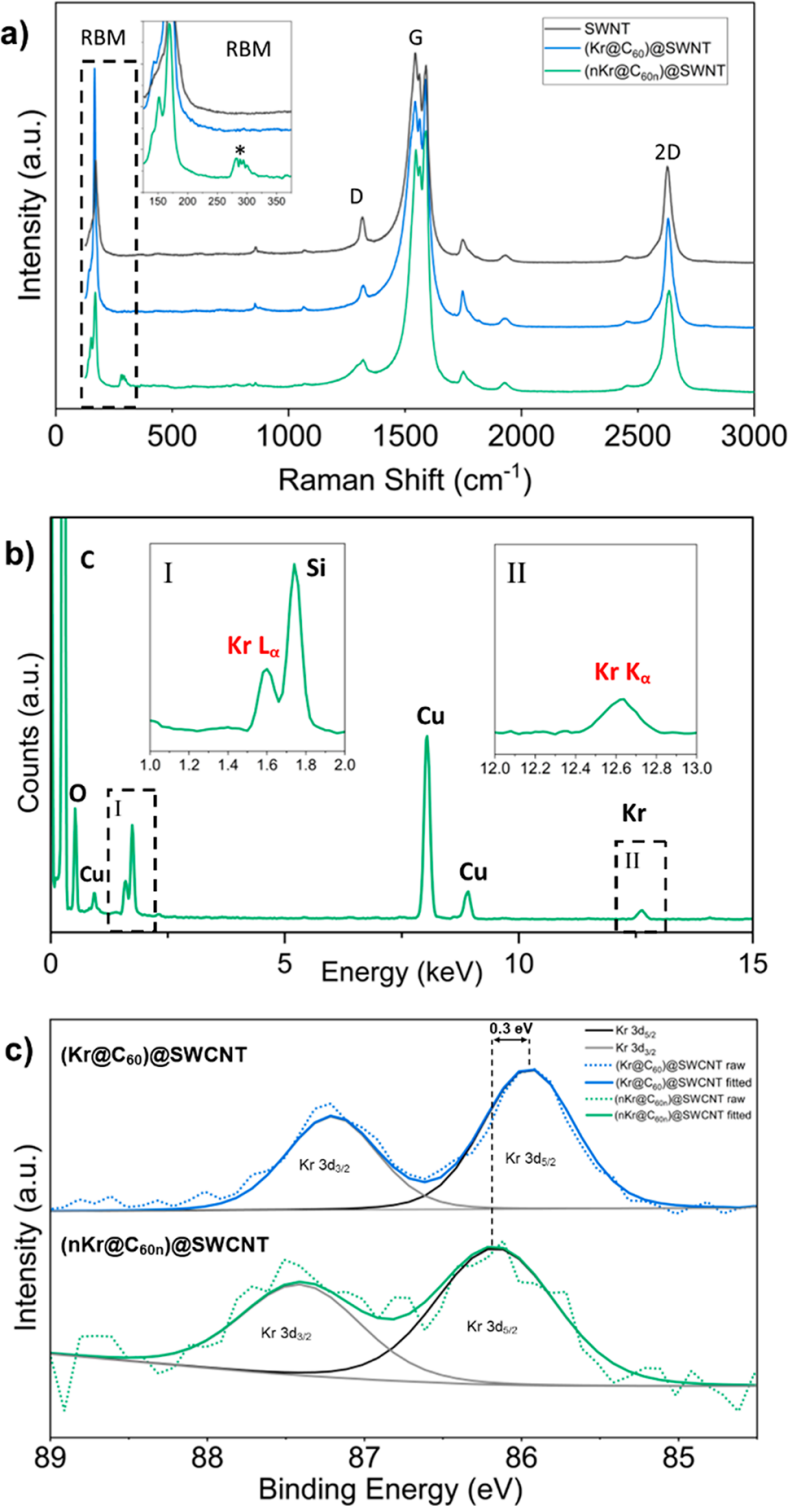
(a) 660 nm resonance Raman spectra of empty metallic SWCNT (gray), (Kr@C_60_)@SWCNT (blue), and (*n*Kr@C_60*n*_)@SWCNT (green), highlighting enlarged RBM, D, and 2D bands (inset). The additional RBM associated with the formed nested nanotubes after thermal treatment is marked with an asterisk. Spectra have been normalized to the intensity of the SWCNT G-band and offset on the *y*-axis for visual clarity. (b) EDS spectrum for (*n*Kr@C_60*n*_)@SWCNT with enlarged Kr Lα (I, 1.6 keV) and Kr Kα (II, 12.6 keV) peaks inset. Additional fluorescent signals were attributed to O and Cu from the support film and TEM grid, with Si from the glass ampule used during SWCNT filling.(c) XPS spectra of the Kr 3d environment for (Kr@C_60_)@SWCNT (blue) and thermally processed (*n*Kr@C_60*n*_)@SWCNT (green). Wide scan XPS spectra are shown in Figure S12.

Figure 5a compares the 660 nm resonance Raman spectra of empty metallic SWCNT (grey), peapod (Kr@C_60_)@SWCNT (blue), and thermally annealed (*n*Kr@C_60*n*_)@SWCNT (green) and provides details of the integrity of the host and nested carbon nanotubes. Post encapsulation of Kr@C_60_ in SWCNT, and post heat treatment to form (*n*Kr@C_60*n*_)@SWCNT, no significant shift in the position of the SWCNT G-band occurred, indicating no charge transfer between guest Kr@C_60_ molecules and the host nanotube.

This supports the expectation that delivered Kr atoms do not interact electronically with the nanotube containers. Post encapsulation of Kr@C_60_, a 4.7 cm^–1^ red shift in the principle SWCNT radial breathing mode (RBM) was observed, which is attributed to an expansion of the nanotube to maximize favorable van der Waals interactions with the guest fullerene molecules.^30^

Further, post heat treatment to form (*n*Kr@C_60*n*_)@SWCNT yielded another set of D and 2D bands (red-shifted relative to the corresponding bands of the host SWCNT), and another series of RBMs centered at ∼293 cm^–1^, consistent with the formation of nested nanotubes of diameters ∼0.83 nm.^31^

Similar observations were noted in the 532 nm resonance Raman spectra of semiconducting SWCNT (Figure S9), including tentative evidence for an additional RBM at 314 cm^–1^, indicating a nested nanotube of diameter ∼0.77 nm, concordant with direct space imaging by AC-HRTEM (Figure 3c). The resonance Raman spectra of both empty and Kr-filled peapods following thermal coalescence were similar, indicating that endohedral Kr does not affect the C_60_ coalescence and annealing mechanism (Figures S10 and S11). This confirms that thermal coalescence occurred via carbon cage rearrangement, without openings through which encapsulated Kr atoms could escape. Indeed, Stone–Wales rearrangements during thermal processing do not lead to cage opening, and hence retention of the encapsulated Kr atoms is anticipated, even at elevated temperature, as confirmed by EDS analysis of (*n*Kr@C_60*n*_)@SWCNT (Figure 5b).

Figure 5c presents X-ray photoelectron spectroscopy (XPS) data for pristine and thermally polymerized (Kr@C_60_)@SWCNT. Pristine peapod material (blue) exhibited peaks at 85.9 and 87.2 eV attributable to Kr 3d_5/2_ and 3d_3/2_ photoelectron lines, respectively, which upon thermal processing to form Kr atom chains (*n*Kr@C_60*n*_)@SWCNT (green) shifted to a higher binding energy by 0.3 eV. In this context, it is noted that Kr intercalated between graphitic layers exhibited a 3d_5/2_ photoelectron line at ∼87 eV,^32^ while the equivalent line for free Kr gas lies at 93.8 eV.^33^

This trend in increasing binding energy from Kr@C_60_ (zero degrees of translational freedom) to Kr gas (three degrees of translational freedom) (Figure S13 and Table S1) further confirms that as Kr atoms are released and become freer to translate along the C_60*n*_ nested nanotubes, Kr transitions toward a gaseous, less constrained state post thermal annealing, as observed in HAADF-STEM imaging. When comparing to XPS studies of other noble gases, it has been reported that the binding energy of Ar 2p electrons shift down by 1.7 eV upon immobilization from the free gas, consistent with this proposition.^34^

### *In Situ* release of Kr Atoms: Electron-Beam-Induced Coalescence of Kr@C_60_

Thermal release of Kr atoms from carbon cages has enabled the study of their dynamic behavior by TEM. Indeed, *in situ* electron-beam-mediated release of Kr, captured by time-resolved TEM imaging, facilitates tracking Kr positions and dynamics with spatiotemporal continuity. The 80 keV electron beam is an ideal probe, as energy transfer is below the threshold for carbon atom displacement in SWCNT, but above that for C_60_,^35^ such that Kr@C_60_ undergoes controlled coalescence while SWCNT remains virtually intact.

**Figure 6:**
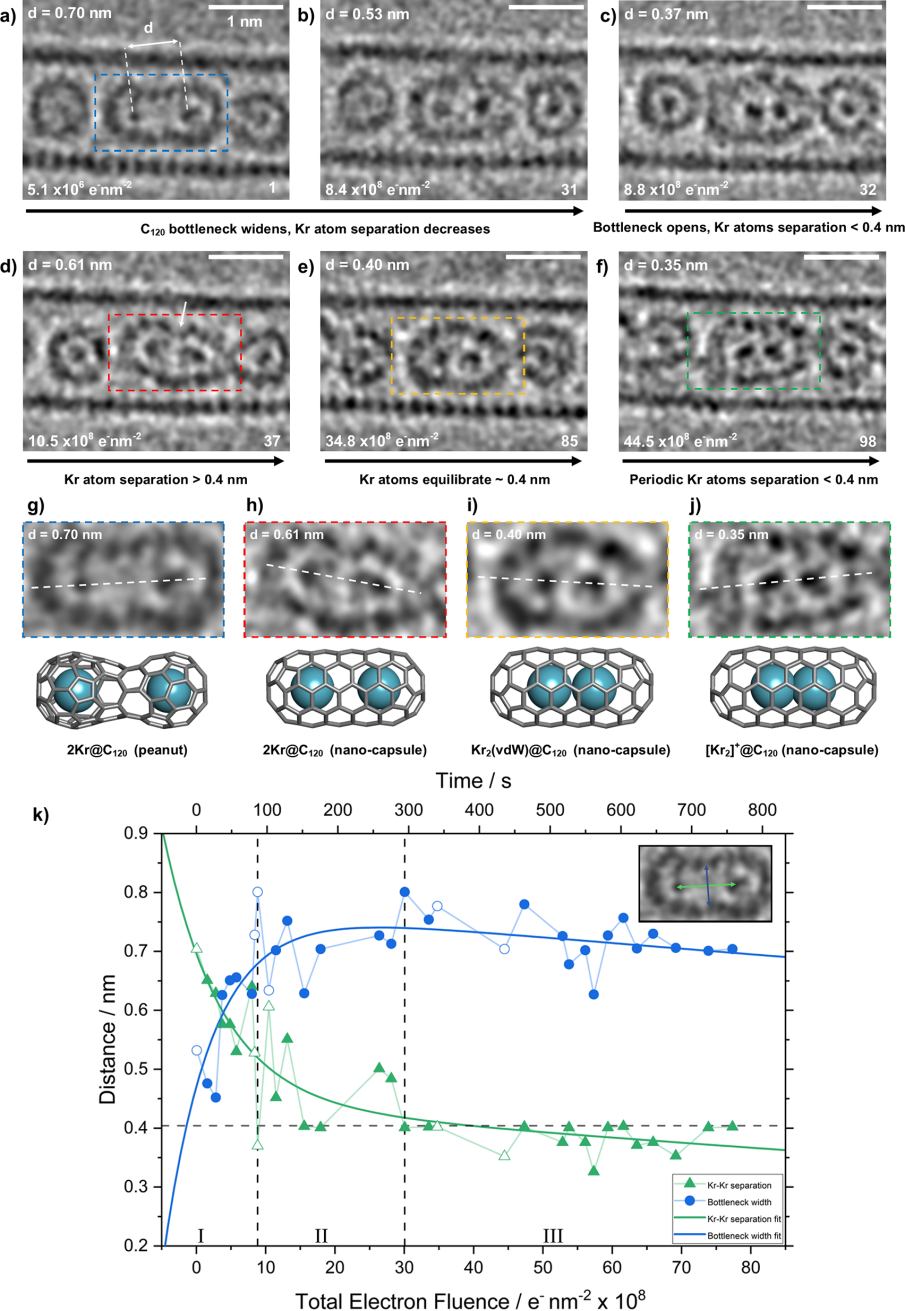
(a-f) Time-series AC-HRTEM images (80 kV; 1 × 10^7^ e^–^ nm^–2^ s^–1^) illustrating the latter stages of coalescence of two Kr@C_60_ molecules encapsulated within a SWCNT. Total electron fluence and frame number for each image is shown at the bottom of each panel, with Kr–Kr separations noted at the top left. (g–j) Enlarged views of (a), (d), (e), and (f), respectively, and accompanying structural models showing the relative position and bonding state of the Kr atoms. (k) Kr–Kr separation (green triangles) and C_120_ bottleneck width (blue circles) for this time series, as a function of increasing time or electron fluence. Unfilled data points correspond to Figure 6a–f. Fitted curves for each data set are shown. The horizontal dashed line indicates the theoretical Kr–Kr van der Waals separation. Statistical data corresponding to dimerization event frequency is shown in Table S2.

The acquisition of sequential images from a region of (Kr@C_60_)@SWCNT under constant electron flux provided for a direct investigation of the dynamic processes between two Kr atoms coencapsulated within a fused C_120_ dimer (Movie S1, cropped and stabilized in Movie S2). Figure 6a–f presents a representative selection of these time-series images charting the latter stages of coalescence of adjacent Kr@C_60_ molecules, recorded at 80 kV under a constant electron flux of 1 × 10^7^ e^–^ nm^–2^ s^–1^. Indeed, due to the stochastic nature of electron-beam-induced reactions, a 2Kr@C_120_ “peanut” intermediate was imaged at the start of this series, formed during the search and focusing stage before time-series acquisition (Figure 6a,g) (see Table S2 in Supporting Information for information on the number of events observed). The peanut annealed with time to form a C_120_ nanotube-type capsule, allowing the free interaction of guest Kr atoms in one dimension, following which Kr···Kr separations (*d*_Kr–Kr_) could be determined via intensity profiling and attributed to particular Kr_2_ bonding states. Figure 6k presents a plot correlating *d*_Kr–Kr_ and C_120_ bottleneck width as a function of electron fluence (and equivalent elapsed time) over the course of this time series. Separations corresponding to Figure 6a–f are highlighted, emphasizing the variation in Kr_2_ bonding states. This plot indicates three distinct regimes corresponding to the separation of Kr···Kr while experiencing constriction, followed by heavily damped free translation as the Kr atoms were released, and then closer Kr bonding as a more settled, stable configuration became established. During Kr restriction by the C_120_ peanut bottleneck (Regime I), the Kr···Kr separation was found to decrease continuously, initially from 0.70 to 0.53 nm (Figure 6a,b) as the e-beam drove the widening of the peanut (2Kr)@C_120_ bottleneck (see Figure S14 for data handling and treatment of errors). With increasing fluence, the bottleneck widened sufficiently to form a nested (2Kr)@C_120_ nanocapsule structure with a rapid decrease in *d*_Kr–Kr_ from 0.64 to 0.37 nm in ∼8 s, indicating a sharp transition toward free Kr atom translation along the nanotube axis (Regime II). For example, representative images in Figure 6c–e illustrate distinct separations of 0.37, 0.61, and 0.40 nm, respectively.

Kr atoms encapsulated within such C_120_ nanocapsules showed increased motion during image acquisition when compared to Kr@C_60_, as evidenced by noncircular atomic contrast (e.g. Figure 6 c,e,f) representative of the weighted average of atom positions during exposure. The difference in *d*_Kr–Kr_ could be as large as 0.1 nm, effectively highlighting the short lifetimes of <0.4 nm separations. Hence, the measurement of *d*_Kr–Kr_ via intensity profiling represents the average separation captured during exposure.

Nevertheless, careful observation demonstrated distinct Kr–Kr separations alternating between extremes of ∼0.6 and ∼0.4 nm, respectively, in the manner of a highly damped oscillation, consistent with distinct states attributable to nonbonded Kr···Kr and a van der Waals Kr_2_ dimer, respectively (Figure 6h,i). Eventually, the Kr atom pair equilibrated (Regime III) to the van der Waals bonded dimer separation, while occasionally decreasing to between 0.32 and 0.38 nm, indicative of stronger Kr–Kr bonding (Figure 6f,j). Figure 6d,h revealed slight distortion to the C_120_ nanocapsule (arrowed), consistent with the theoretical suggestion that a perfect (5,5) C_120_ nanotube is not necessarily formed,^36^ while noting the capsule, in this state, did not inhibit Kr atom bonding.

**Figure 7:**
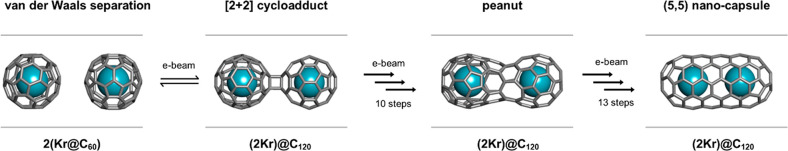
Adaptation of the Osawa–Tománek mechanism^37^ for e-beam coalescence of 2(Kr@C_60_) encapsulated within SWCNT. The mechanism proceeds via a reversible [2 + 2] cycloaddition, followed by a retro [2 + 2] and 22 subsequent Stone–Wales rearrangements, leading to the formation of a straight-walled C_120_ nanocapsule. Kr atoms are constrained in the [2 + 2] and peanut intermediates, requiring complete annealing to fully integral nested SWCNT in which coencapsulated Kr atoms can translate. The endohedral species is expected to have no effect on this mechanism and to remain entrapped during the process (Supporting Information).

The observed change in Kr separation can be rationalized by adapting the Osawa–Tománek (OT) mechanism for C_60_ coalescence^37^ to the transformation of 2(Kr@C_60_) to (2Kr)@C_120_ (Figure 7). Each Kr atom is confined to its host C_60_ cage as dumbbell-shaped (2Kr)@C_120_ [2 + 2] cycloadducts form and remain immobilized after the formation of the (2Kr)@C_120_ peanut structure, as the bottleneck between the cages is too narrow for Kr atoms to transit (∼0.19 nm opening) (Figure 6a,b). It is only in the final part of the transformation to form the (2Kr)@C_120_ nanocapsule structure that the bottleneck becomes sufficiently wide for the transit of Kr atoms (∼0.4 nm opening) (Figure 6c), in turn providing for direct observation of coencapsulated Kr atoms free to interact within the C_120_ cavity.

It is noted that the close-packed atomic spacing of Kr in face-centered cubic (fcc) crystallites was previously determined to be 0.399 nm by X-ray diffractometry.^38^ This is commensurate with the van der Waals Kr_2_ dimer separation observed directly here by TEM after stabilization of Kr atom pairs (Regime III; Figure 6e,i), being the favored configuration distinct from noninteracting gaseous species. Several instances of Kr–Kr atom separation significantly below 0.4 nm were observed during Regime III, e.g., down to 0.33 nm. The lifetime of each of these separations was again at least on the scale of the 0.5 s exposure time for data acquisition, i.e., much longer than expected for a transient minimum for a neutral van der Waals dimer where strong repulsion due to the Pauli exclusion principle would act quickly to re-establish the energetically favored 0.4 nm Kr atom separation. Hence, the presence of relatively long-lived, <0.4 nm Kr–Kr separations is consistent with the formation of a transient covalent bond, i.e., in the form of a cationic dimer [Kr_2_]^+^. Further, it is interesting to note that Figure 6c, demonstrating close separation interaction of Kr atoms free of constriction, shows ∼0.15 nm of clear space (when accounting for the van der Waals radii of both C and Kr) between the two Kr atoms and the end of the host capsule. This confirms that short-separation Kr–Kr interactions occur free of constriction from the encapsulating C_120_ capsule. This form of [Kr_2_]^+^ has been studied computationally and identified by laser spectroscopy for gaseous Kr, with bond lengths ranging from ∼0.28 to ∼0.41 nm depending on electronic state.^39,40^

It is noted that the predominant mechanism of energy transfer for encapsulated species in SWCNT is typically direct knock-on damage (DKO), whereby kinetic energy (momentum) is transferred directly from an incident highly energetic electron to the nucleus of a sample atom. An 80 keV electron beam is strongly ionizing; however, ionization damage is unlikely due to the highly conducting nature of the host nanotube, as C_60_^+^ will be quenched rapidly, with the highest occupied molecular orbital (HOMO) for C_60_ positioned below the Fermi level for metallic SWCNT or midgap energy for semiconducting SWCNT,^41^ while also noting it has been proposed that an ionization process might initiate the onset of C_60_ coalescence.^42^

However, an 80 keV electron could interact with and eject any Kr electron, accompanied by the emission of an X-ray photon following energy relaxation, ultimately forming valence-hole Kr^+^ in its lowest energy state. A freely translating Kr^+^ cation can then bond covalently to a coencapsulated neutral Kr atom, denoted (Kr^+^|Kr)@C_120_ to form [Kr_2_]^+^@C_120_ with an observed bond length ranging between 0.33 and 0.38 nm and being sufficiently long-lived to be detected on the time scale of TEM imaging in a highly constrained environment (Figure 6c,f). It is noted that Kr 4s and 4p orbitals are low lying relative to C_60_ (>10 eV from a consideration of electron affinity), and so neutralization of [Kr_2_]^+^ by SWCNT via C_120_ could be considered an energetically favorable pathway. However, theoretical studies of Kr@C_60_ have revealed only very slight hybridization between Kr 4p and C_60_ molecular orbitals, and none for the case of Kr 4s.^43,44^ Hence, the poor overlap between Kr and C_60_ (and by implication C_120_) electronic systems extends the lifetime of [Kr_2_]^+^ toward the 0.5 s acquisition time utilized in TEM. Eventual neutralization and subsequent bond dissociation of [Kr_2_]^+^ via the host SWCNT would result in repulsion between two now neutral Kr atoms, returning the system to the preferred Kr_2_ dimer at 0.40 nm separation (Figure 6e), i.e., consistent with an *oscillation* between van der Waals Kr_2_ dimer and [Kr_2_]^+^ bonding upon free translation, for the lifetimes as observed in TEM.

### *In Situ* Manipulation of Chains of Kr Atoms: Electron-Beam-Induced Annealing of Defects in Nanotubes

Localized electron beam annealing of structural defects in nested nanotubes formed by thermal coalescence of Kr@C_60_ allowed the formation and investigation of longer Kr atomic chains, thereby expanding the study of Kr–Kr interactions beyond simple dimers.

**Figure 8:**
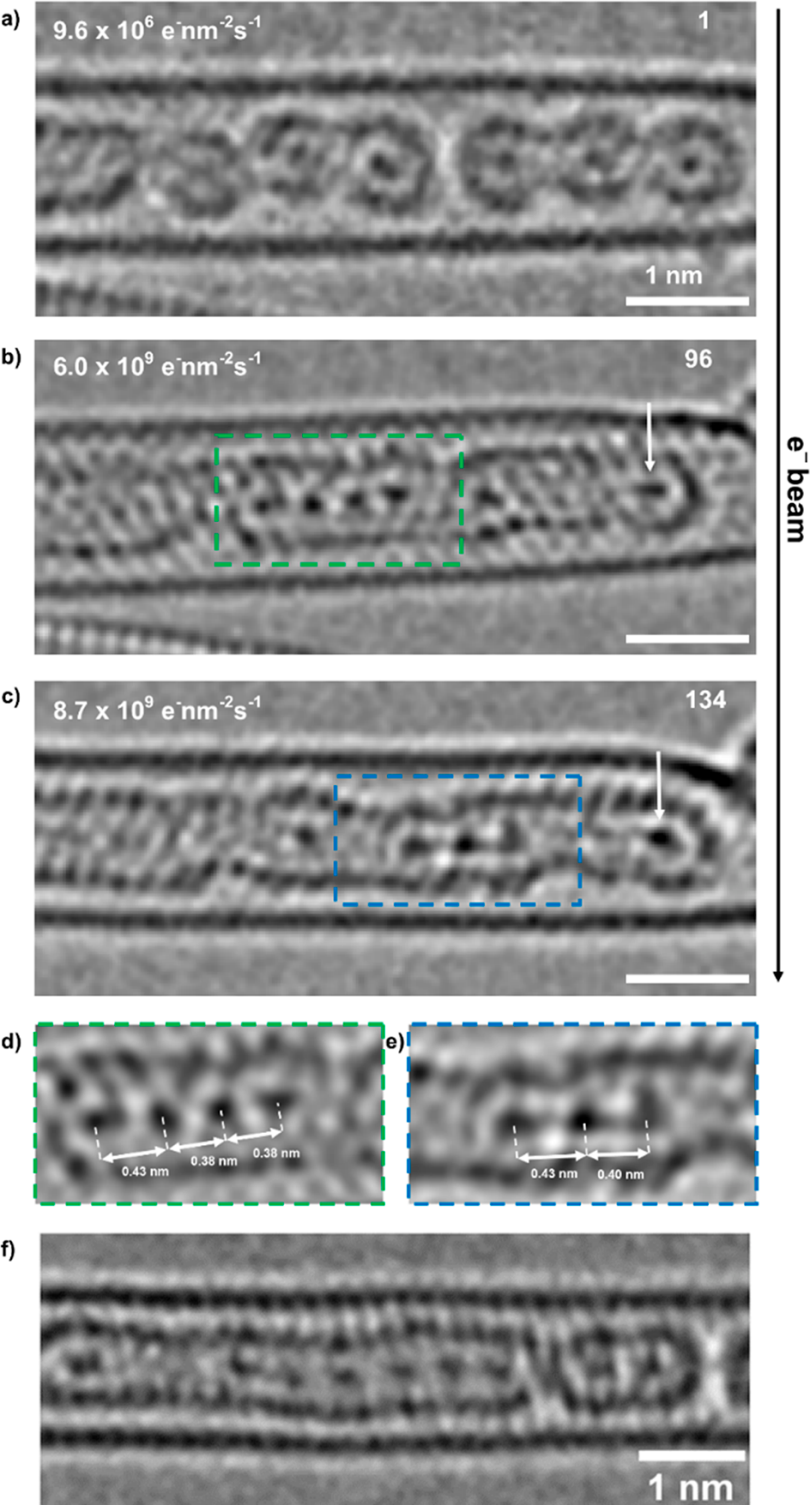
(a-c) Time-series AC-HRTEM images (80 kV; 4.3 × 10^7^ e^–^ nm^–2^ s^–1^) charting the electron-beam-induced coalescence of six thermally precoalesced Kr@C_60_ molecules, highlighting the one-dimensional translation and bonding of the cluster of six Kr atoms. (d, e) Expanded views of (b, c), respectively, with Kr–Kr separations explicitly shown. (f) AC-HRTEM image of a thermally formed nested nanotube with all seven guest Kr atoms visible, six of which form a chain with spacings ranging 0.46–0.56 nm.

By way of illustration, Figure 8a–c presents a time-lapse series from a short region of partially thermally coalesced Kr@C_60_ molecules where six Kr atoms remained distinct, as the electron beam annealed nested nanotube structural defects, thus removing barriers to Kr translation (Movie S3). In particular, Figure 8b shows a chain of four interacting Kr atoms, pinned to the left-hand side of the nanotube, with *d*_Kr–Kr_ spacings of 0.43, 0.38, and 0.38 nm respectively (Figure 8d), indicative of a pinned terminal Kr atom attached to a Kr_3_ trimer, along with an isolated Kr atom midtube and another pinned terminal Kr atom at the other end (arrowed).

Continued observation (Figure 8c) provided a snapshot of the translation of the Kr_3_ trimer, now midtube, with spacings of 0.43 and 0.40 nm, respectively (Figure 8e), closer to the favored van der Waals separation, with the terminal Kr atoms remaining pinned (arrowed) and the sixth atom moving too quickly to be imaged. It is considered that the higher surface area associated with the curvature of the end caps contributes to the pinning of terminal Kr atoms.

To demonstrate the potential of this methodology to image longer atomic chains, Figure 8f is illustrative of a chain of six Kr atoms, with a terminal Kr atom pinned at the right-hand side (arrowed). In this case, larger spacings were identified between the Kr atomic chains ranging from 0.46 to 0.56 nm, indicative of more weakly bound associations of these atoms, providing a tentative indication of an intermediate state of Kr before transitioning to a 1D gas.

## Discussion

Observation of atomic dynamics using transmission electron microscopy provides an opportunity to investigate chemical processes at the nanoscale. Nanomaterials are known to behave differently to the bulk phase, and extreme confinement resulting from nanotube encapsulation forces a highly constrained environment on the material, which can lead to a greater understanding of the fundamental properties of a material and to the discovery of how atoms behave at the nanoscale. In this context, this protocol based on endohedral fullerenes as carriers of atoms and the formation of nested nanotube vessels for the delivery, confinement, and direct observation of single noble gas atoms, dimers, chains, and 1D gas raises interesting fundamental questions about the interactions that govern chemical reactivity and the nature of matter when confined at the nanoscale to 1D. TEM has previously been utilized to study 1D atomic chains of carbon,^45^ gold,^46,47^ iodine,^48^ and ionic crystals.^49^

Here we have shown that the electron beam or heat may be used to control the formation of short capsules or longer nested nanotube containers, respectively, appropriate for the direct observation of short associations of Kr atoms. The resultant containers, once fully developed, provide excess space for free translation of the delivered guest atoms (i.e., 2/3 occupied by solid packing of Kr van der Waals spheres and 1/3 free space (Figure S8)), hence allowing Kr to return toward a gaseous state. An average relative intensity of ∼0.66 was measured for mobile versus stationary Kr atoms, commensurate with this proposition. It is noted that the van der Waals diameter of Kr is such that it fits the 0.7 nm diameter of the internal vessel so that Kr atoms cannot pass each other, akin to the beads of an abacus, similar in structure to previously studied 1D Tonks–Girardeau gases.^50,51^

Intriguingly, it is noted that the theoretical velocity of gaseous Kr atoms in 1D is ∼170 ms^–1^ at room temperature (eq S2); however, a diffuse signal can be detected in well-annealed nested nanotubes by both HAADF-STEM imaging and STEM-EELS mapping. Theoretical consideration of Kr gas in 3D at standard temperature and pressure yields an average Kr···Kr atomic separation of ∼3 nm (eq S3) with a mean free path of ∼57 nm (eq S4). However, the highly efficient 1D packing of Kr, where atoms cannot pass each other, yields an average atomic separation of ∼0.6 nm. This reduction in atomic separation relative to the free 3D gas, upon constriction to 1D, drastically decreases the mean free path of Kr and radically changes the gaseous behavior. The pressure exerted by gaseous Kr on the end caps of the nested nanotube container is ∼150 MPa (eq S5), representative of the extreme confinement and high density of Kr gas. We have confirmed the presence of completely delocalized, freely translating Kr atoms within the nested nanotube, providing the physical realization of one-dimensional gas models hypothesized in numerous theoretical studies, in principle giving insights into physical phenomena such as heat conduction and diffusion or hydrodynamics.^52−56^ The heating time during thermal coalescence directly affects both the degree of polymerization and number of defects within nested nanotubes, in principle giving control over both the length and pressure of the 1D Kr gas, by altering the available space for free translation.

The evidence suggests that visible, stationary atom chains may be associated with residual pinning points, by way of transient stabilization on the time scale of data acquisition. The intermediate condition identified between binary pairs of atoms in short capsules and longer atomic chains in extended nested nanotubes, i.e., the elongation of bonding between neighboring Kr atoms, provides a tantalising glimpse of the intermediate state between an atomic chain and a 1D gaseous state.

Detailed investigation of the dynamics of Kr–Kr atom pairs reveals van der Waals dimer formation by way of preferred spacing, while energetic fluctuations indicate the transient formation of covalent [Kr_2_]^+^, induced by the electron beam. Conversely, for the case of longer chains such as Kr_6_, derived from thermal and electron beam processing, the evidence suggests a transition to a more loosely bonded state consistent with the onset of a transition to the gaseous phase, as shaped by the container.

Ultimately, this emphasizes the stochastic behavior of the noble gas atoms under investigation. The highly confining nature of the nested nano test tubes, combined with the very high density of Kr atom packing, limits the atoms to a single translational degree of freedom along the nanotube axis. This radically changes the atomistic behavior toward that of a highly compressed gas with no degrees of freedom for dimensional change.

In the absence of a pinning point, the associations of Kr atoms are still too mobile, resulting in single-atom contrast smearing in HAADF-STEM images.^57^ Whether or not such fast-translating atoms move as connected short chains or as individual atoms, and their states, remains unknown. This may be addressed in the future through use of higher frame rate imaging electron cameras combined with low temperature.

## Conclusions

Carbon nanotubes provide excellent platforms for imaging and analysis, allowing high-resolution investigations into the atomic world. Among chemical elements, the noble gases have been the most elusive for dynamic investigations at the atomic scale,^22,23,25^ which stimulated the development of a molecular system for the delivery and direct observation of krypton atom dynamics in direct space and real time.

Entrapment of individual Kr atoms in fullerene cages C_60_ (Kr@C_60_), followed by encapsulation into carbon nanotubes yielded the nanoscale system (Kr@C_60_)@SWCNT in which Kr atom positions and chemical identity were confirmed by TEM imaging and spectroscopy. Interactions and bonding between Kr atoms were examined with spatiotemporal continuity, tracking changes in real time at the atomic level.

Application of the electron beam facilitated fullerene coalescence and allowed the formation of a van der Waals Kr dimer, with the occasional reversible formation of a covalent cationic dimer [Kr_2_]^+^ being identified. Thermal annealing *ex situ* formed nested nanotubes, in which the local environment around Kr atoms was found to be essential in controlling their translation. In long, well-annealed sections, delocalized Kr atom contrast was visible in HAADF-STEM and STEM-EELS mapping, confirming a 1D gas-like state of the noble gas within the nanotube. Combination with electron beam processing facilitated the formation of short atomic chains Kr_*n*_ (*n* ≤ 6), with elongated bonding states, evidencing the transition to a highly compressed 1D gas. This gaseous state of matter is stable under ambient conditions, facilitating future opportunities to probe 1D gases by a variety of analytical techniques.

This methodology builds on the concepts of the atom-forge^21^ and time-resolved TEM^1^ and hence offers an exciting array of opportunities for the investigation of selected atom combinations, enabling a wide range of chemical processes to be observed directly at the atomic scale and therefore providing a paradigm for studying chemistry at the fundamental level.

## Methods

### Materials

SWCNT (P2-SWCNT, arc discharge, Carbon Solutions, USA) was annealed in air at 600 °C for 17 min to ensure the complete removal of the end caps and any residual amorphous carbon. Buckminsterfullerene C_60_ (Nano-C, USA) was used without further purification. Kr@C_60_ was synthesized as described in ref (26). C_60_ and Kr@C_60_ were filled into an opened SWCNT of average diameter ∼1.4 nm, via sublimation, by sealing in Pyrex ampules under vacuum (10^–5^ mbar) and heating at 550 °C for 72 h, to form C_60_@SWCNT and (Kr@C_60_)@SWCNT, respectively. *Ex situ* thermal coalescence of C_60_@SWCNT and (Kr@C_60_)@SWCNT was achieved by sealing the respective powders in quartz ampules under an argon atmosphere at 0.6 bar, then heating at 1200 °C for 6 h. Prepared materials were dispersed in isopropanol using an ultrasonic bath and drop-cast directly onto lacey-carbon-coated copper TEM grids (Agar Scientific) for characterization.

### TEM Data Acquisition

Aberration-corrected HRTEM imaging at Ulm University was performed using a dedicated sub-ångström low-voltage electron microscopy (SALVE) instrument based on a Thermo-Fischer Themis platform, equipped with dedicated chromatic and spherical (C_c_/C_s_) aberration correctors developed by CEOS. The SALVE instrument is fully corrected for fifth-order axial geometric aberrations (including C_s_ and C_5_), for third-order off-axial geometric aberrations, and for first-order chromatic aberrations (C_c_). The microscope was operated at 80 kV. Images were acquired using a Gatan Ultrascan 1000 XP with exposure times of 0.25 or 0.5 s (×2 binning; 1024 × 1024 image pixels). A low electron flux (∼10^5^ e^–^ nm^–2^ s^–1^) was used to focus close to regions of interest, in order to minimize onset of beam-induced transformations. An electron flux of approximately 10^7^ e^–^ nm^–2^ s^–1^ was used for image series acquisition (5 s interval frame rate) and to induce chemical transformations.

STEM-EDS mapping together with HAADF imaging was performed on a Thermo Fisher Talos 200X instrument operated at 120 kV equipped with a windowless four-segment SuperX EDS detector.

Complementary high-resolution TEM imaging was performed on a JEOL 2100F FEG-TEM microscope operated at 200 kV equipped with a Gatan K3-IS camera, and an Oxford Instruments XMax 80 detector and INCA X-ray microanalysis software were used for EDS investigations.

Additional scanning transmission electron microscopy (STEM) observations were performed at 60 kV acceleration voltage on a Nion UltraSTEM 100 at the SuperSTEM laboratory, Daresbury, UK. This microscope is equipped with a fifth-order probe aberration corrector enabling a probe size of ∼0.09 nm at 60 kV with a convergence semiangle of 31 mrad and a probe current of 30 pA in the conditions used for these experiments. High-angle annular-dark-field (HAADF) images were recorded using a detector with a semiangular range of 85–195 mrad. Electron energy loss spectra were acquired on a Gatan Enfina spectrometer, modified with high-stability electronics for improved performance and retrofitted with a Quantum Detectors Merlin EELS hybrid pixel camera. The EELS collection semiangle was 36 mrad, with spectrum images acquired in “event-streamed” mode, whereby thanks to the minimal sample drift of the instrument (less than 0.5 nm/hour in the experimental conditions), consecutive spectrum images with short pixel dwell times (2 ms/pixel) are accumulated until a sufficient signal is acquired, while reducing noise thanks to the multiple acquisitions. Chemical maps were generated by integration of the relevant ionization edges, as described in the text, after subtraction of the decaying background using a standard power law function. The data was denoised using principal component analysis, as implemented in Gatan Microscopy Suite 3.5 (GMS3.5), with residuals carefully inspected to avoid the introduction of artifacts. Of note, the near-perfect Poisson-limited nature of data acquired on next-generation hybrid pixel detectors lends itself particularly well to such processing with limited artifact generation.^58^

### TEM Image Handling and Analysis

All TEM image analysis was performed using 32-bit raw images. For the presentation of time lapse images as movies, native Gatan.dm3 files were converted to.tif format using GMS3.5 software (with associated transformation from 32 bit to 8 bit image type). Image stacks were processed using ImageJ software (FIJI package)^59^ to enhance contrast and correct for drift (contrast enhancement to 0.35% saturated pixel; FFT band-pass filter of structures between 3 and 40 pixels; images aligned using the StackReg plugin;^60^ cropped and rotated for ease of display). Distance measurements were made using a 5 pixel width linear intensity profile along the Kr–Kr axis, with *d*_Kr–Kr_ determined between intensity minima corresponding to the average central atomic position (see Figure S14).

### Raman Spectroscopy

Micro Raman spectroscopy was performed using a HORIBA LabRAM HR Raman microscope. Spectra were acquired using either 532 or 660 nm lasers, a 100× objective, and a 200 μm confocal pinhole. To scan simultaneously a range of Raman shifts, a 600 lines mm^–1^ rotatable diffraction grating along a path length of 800 mm was used. Spectra were acquired using a Synapse CCD detector (1024 pixels), thermoelectrically cooled to −60 °C. In advance of spectral acquisition, the instrument was calibrated using the zero-order line and a standard Si(100) reference band at 520.7 cm^–1^. The spectral resolutions were better than 1.9 and 1.3 cm^–1^ for the 532 and 660 nm laser configurations, respectively.

### X-ray Photoelectron Spectroscopy

XPS was performed using a Kratos AXIS SUPRA PLUS instrument with a monochromatic Al Kα X-ray source (*h*ν = 1486.6 eV) operated at room temperature with 10 mA emission current and 12 kV anode potential. The electron collection spot size was ca. 700 × 300 μm^2^. A pass energy of 160 eV was used for the survey scans and 20 eV for the high-resolution scans. Spectra were converted into VAMAS format for further analysis and processed using Casa XPS, software version 2.3.22.

### Computational Calculations

DFT calculations of C_120_ structures were performed using the Q-Chem 5.0 quantum chemistry software package,^61^ using the B3LYP correlation functional and a 6-31G* basis set. Chemical models were made using Avogadro open-source molecular builder and visualization tool, version 1.2.0.^62^

## Supporting Information Available

Further data including TEM, STEM-EDX, EELS, Raman spectroscopy, XPS, TEM measurement methodology, and calculations (PDF)
AC-HRTEM imaging of dynamics of (Kr@C_60_)@SWCNT under 80 kV e-beam (AVI)
AC-HRTEM imaging of Kr dimer formation in Kr_2_@C_120_ (AVI)
AC-HRTEM imaging of dynamics of (nKr@C_60*n*_)@SWCNT under 80 kV e-beam (AVI)

## Supplementary Material

nn3c07853_si_001.pdf

nn3c07853_si_002.avi

nn3c07853_si_003.avi

nn3c07853_si_004.avi
