# Supplementary material for: Atomic-Scale Time-Resolved Imaging of Krypton Dimers, Chains and Transition to a One-Dimensional Gas
Source: ACS Nano. 2024 Jan 22;18(4):2958–71. doi: 10.1021/acsnano.3c07853 (PMC10832048; doi:10.1021/acsnano.3c07853)
Supplement: Supplementary file 1 — nn3c07853_si_001.pdf [file nn3c07853_si_001.pdf]

Supporting Information

Atomic-Scale Time-Resolved Imaging of Krypton Dimers, Chains, and the Transition to a One-Dimensional Gas

Ian Cardillo-Zallo,[1] Johannes Biskupek,[2] Sally Bloodworth,[3] Elizabeth S. Marsden,[3] Michael W. Fay,[4] Quentin M. Ramasse,[5,6] Graham A. Rance,[4] Craig T. Stoppiello,[7] William J. Cull,[1] Benjamin L. Weare,[4] Richard J. Whitby,[3] Ute Kaiser,[2] Paul D. Brown,[8] Andrei N. Khlobystov[1]

[1]Schoolof Chemistry, University of Nottingham, Nottingham NG7 2RD, United Kingdom

[2]Electron Microscopy of Materials Science, Central Facility for Electron Microscopy, Ulm University, Ulm 89081, Germany

[3] School of Chemistry, University of Southampton, Southampton SO17 1BJ, United Kingdom

[4]Nanoscale and Microscale Research Centre, University of Nottingham, Nottingham, NG7 2QL, United Kingdom

[5]SuperSTEM Laboratory, SciTech Daresbury Campus, Daresbury WA4 4AD, United Kingdom

[6]School of Chemical and Process Engineering, and School of Physics and Astronomy, University of Leeds, Leeds, LS29JT, United Kingdom

[7]Centre for Microscopy and Microanalysis, The University of Queensland, Brisbane, Qld 4072, Australia

[8]Department of Mechanical, Materials & Manufacturing Engineering, University of Nottingham, Nottingham, NG7 2RD, United Kingdom

S1. HRTEM image of (Kr@C_{60})@SWCNT 2
S2. AC-HRTEM image of (Kr@C_{60})@SWCNT 2
S3. Calculation of expected Kr EDS abundance 3
S4. STEM-EDS mapping of (Kr@C_{60})@SWCNT 4
S5. Fitted background for EEL spectrum of (Kr@C_{60})@SWCNT 5
S6. HRTEM image of (nKr@C_{60n})@SWCNT 5
S7. Fitted background for EEL spectrum of (nKr@C_{60n})@SWCNT 6
S8. Measurement of 1D Kr gas atom intensity in HAADF-STEM 7
S9. 532 nm resonance Raman spectra of (Kr@C_{60})@SWCNT 9
S10. 532 nm resonance Raman spectra of C_{60}@SWCNT 10
S11. 660 nm resonance Raman spectra of C_{60}@SWCNT 11
S12. Wide scan XPS spectra of (Kr@C_{60})@SWCNT and (nKr@C_{60n})@SWCNT 12
S13. Plot of Kr XPS binding energies with decreasing degrees of freedom 12
Table S1. Data of Kr XPS binding energies with decreasing degrees of freedom 13
S14. Methodology of measurement of d_{Kr-Kr} from AC-HRTEM images 13
Table S2. Statistics of Kr dimerisation events 14
Justification for adaptation of Osawa-Tomanek mechanism of C_{60} coalescence to Kr@C_{60} 14
Calculations 14
Supporting References 16

Supporting Video 1. AC-HRTEM imaging of dynamics of (Kr@C_{60})@SWCNT under 80 kV e-beam
Supporting Video 2. Stabilised imaging of 2Kr@C_{120} capsule from Supporting Video 1
Supporting Video 3. AC-HRTEM imaging of dynamics of (nKr@C_{60n})@SWCNT under 80 kV e-beam

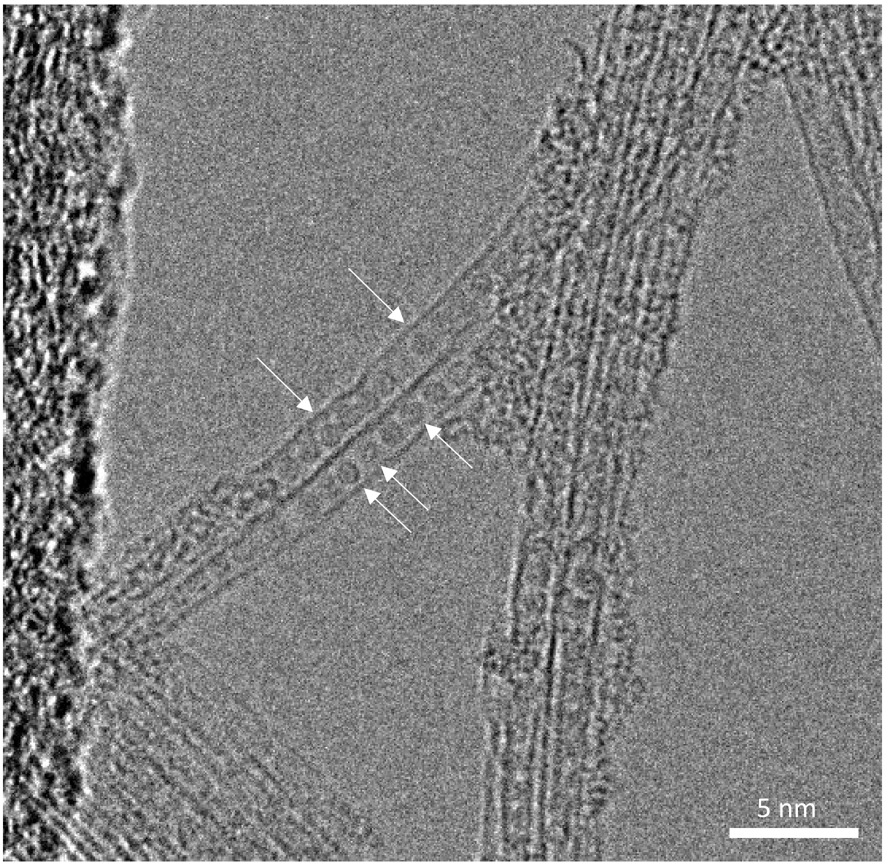

Figure S1. Survey HRTEM image of several bundles of (Kr@C_{60})@SWCNT acquired on a JEOL 2100F TEM operated at 200 kV, using a Gatan K3-IS direct electron detector. The electron flux was 7.5 x 10^3 $e^{-}nm^{-2}s^{-1}$, and the image acquisition time was 0.5 s. In some places individual Kr atoms can be seen (arrowed).

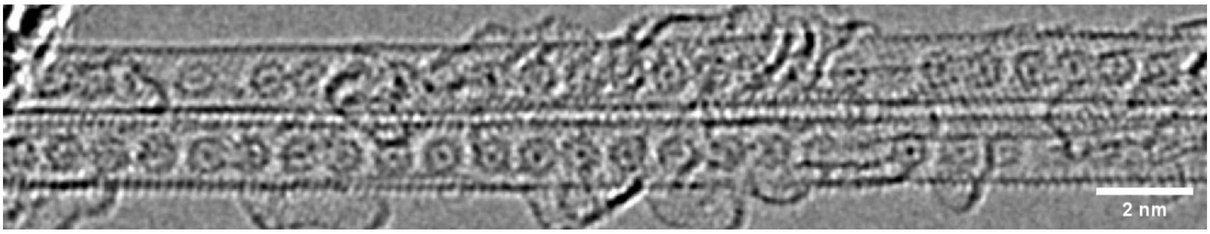

Figure S2. AC-HRTEM image of (Kr@C_{60})@SWCNT acquired at 80 kV on the C_C/C_S corrected SALVE TEM using a Gatan Ultrascan 1000XP camera. A high degree of filling of Kr@C_{60} is observed, whilst noting the two SWCNT shown are contaminated with amorphous carbon.

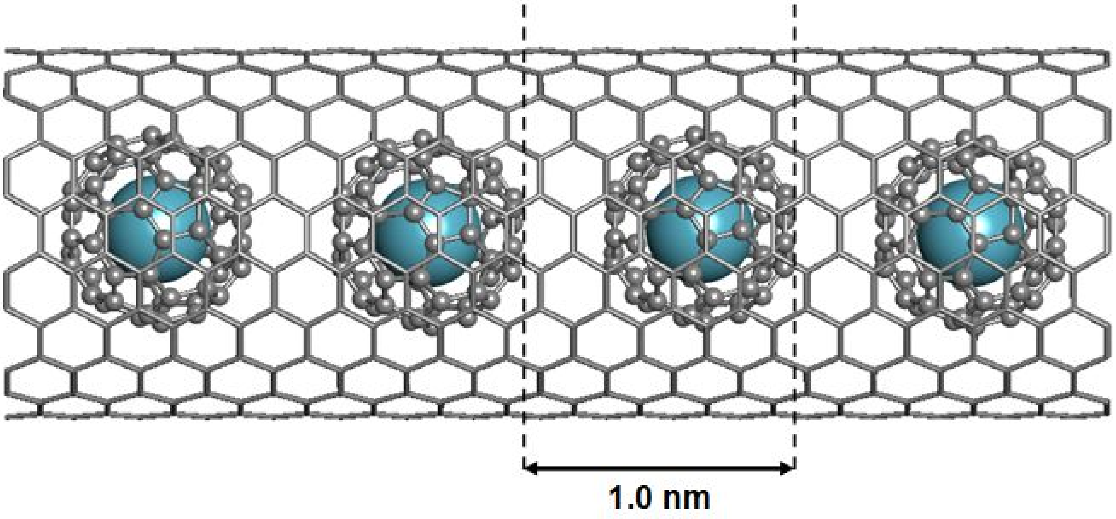

Figure S3. Structural model for (Kr@C_{60})@SWCNT used to calculate the expected abundance of Kr:C via EDS analysis. Kr@C_{60} molecules are spaced at 1.0 nm intervals along the axis of a (10,10) SWCNT.

Kr@C_{60} molecules spaced by 1.0 nm in (10,10) SWCNT (d=1.36 nm).

Number of carbon atom in 1.0 nm length of (10,10) SWCNT = 180

Total number of carbon atoms per 1.0 nm of (Kr@C_{60})@SWCNT = 180 + 60 = 240

Abundance of Kr:C = 1:240 = 0.42 at%

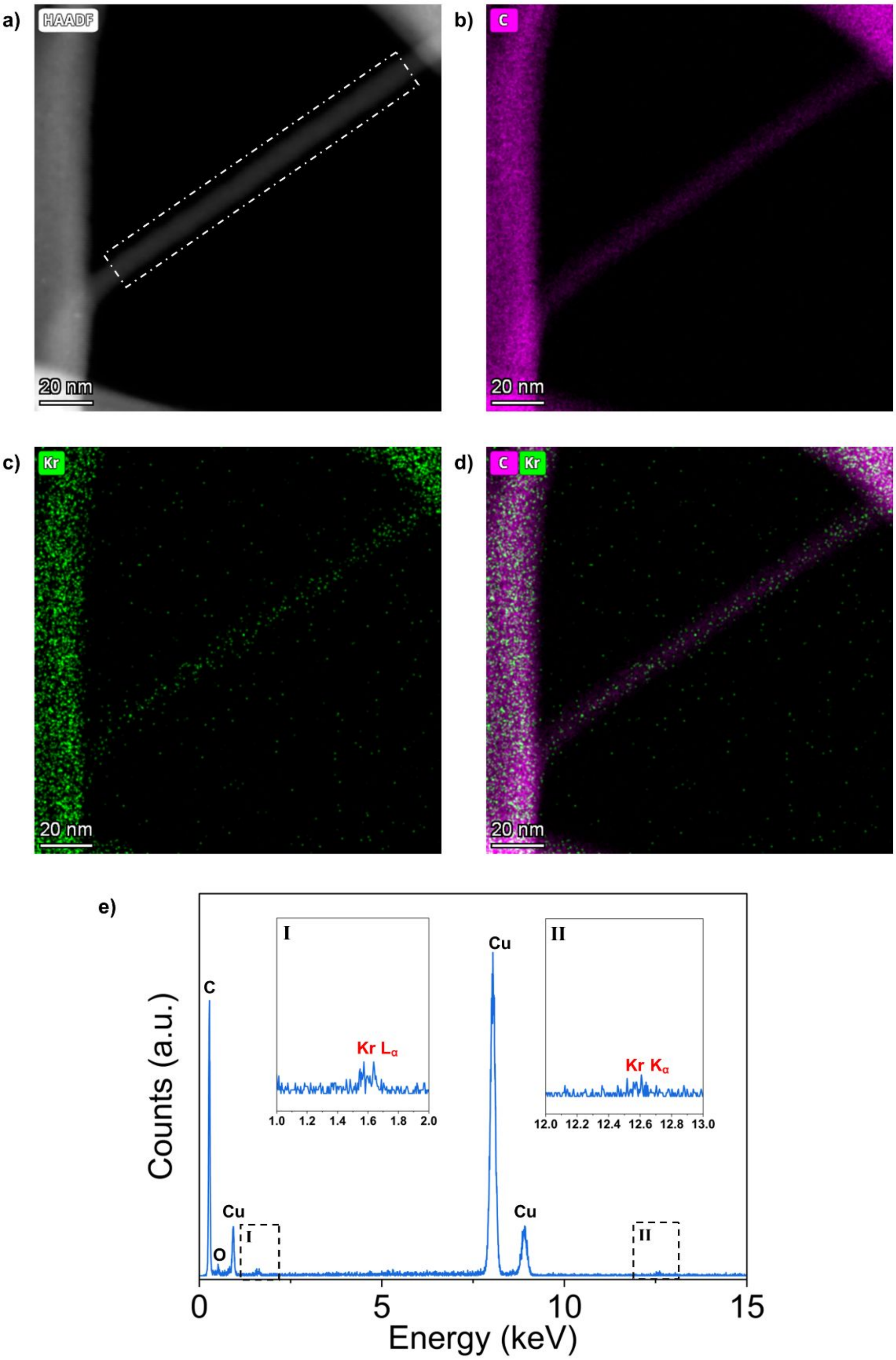

Figure S4. STEM-EDS mapping of (Kr@C_{60})@SWCNT acquired at 120 kV on a Thermo Fischer Talos 200X. a) HAADF-STEM image of a bundle of (Kr@C_{60})@SWCNT. b) Map of carbon (magenta), c) map of krypton (green) and d) overlayed maps of carbon and krypton, showing Kr signal is incident with C signal from SWCNT bundles. e) EDS spectrum from signal acquired in the dashed area shown in a). Additional O and Cu fluorescent signals were attributed to the support film and TEM grid, respectively. The measured abundance of Kr in this area was 0.44 at% relative to C.

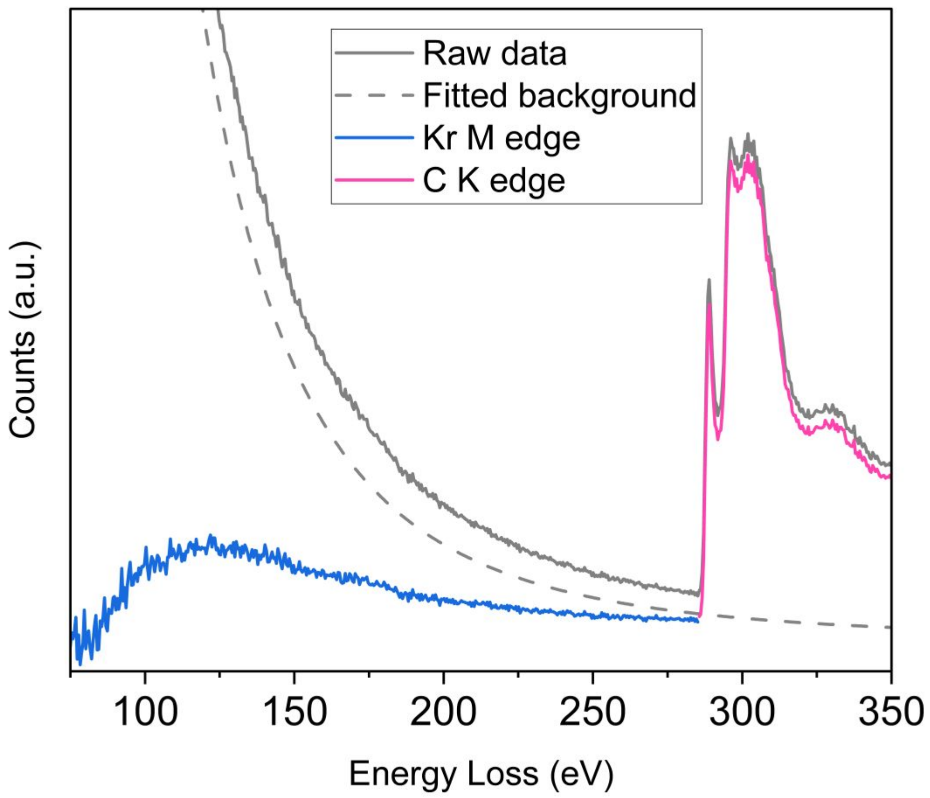

Figure S5. EEL spectrum acquired from integration of signal in green box (Figure 2j), showing raw data and the fitted background model.

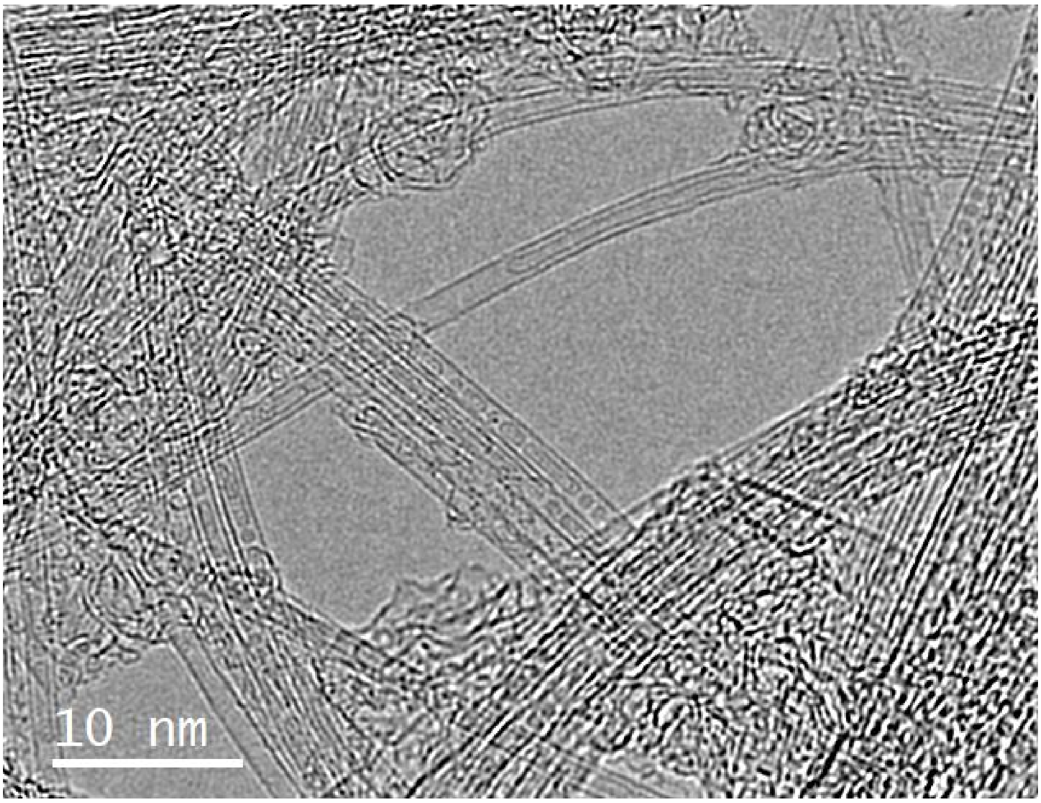

Figure S6. Survey HRTEM image of several bundles of (nKr@C_{60n})@SWCNT acquired on a JEOL 2100F TEM operated at 200 kV, using a Gatan K3-IS direct electron detector. The electron flux was 2.0 x 10^5 $e^-nm^{-2}s^{-1}$, and the image acquisition time was 1.1 s.

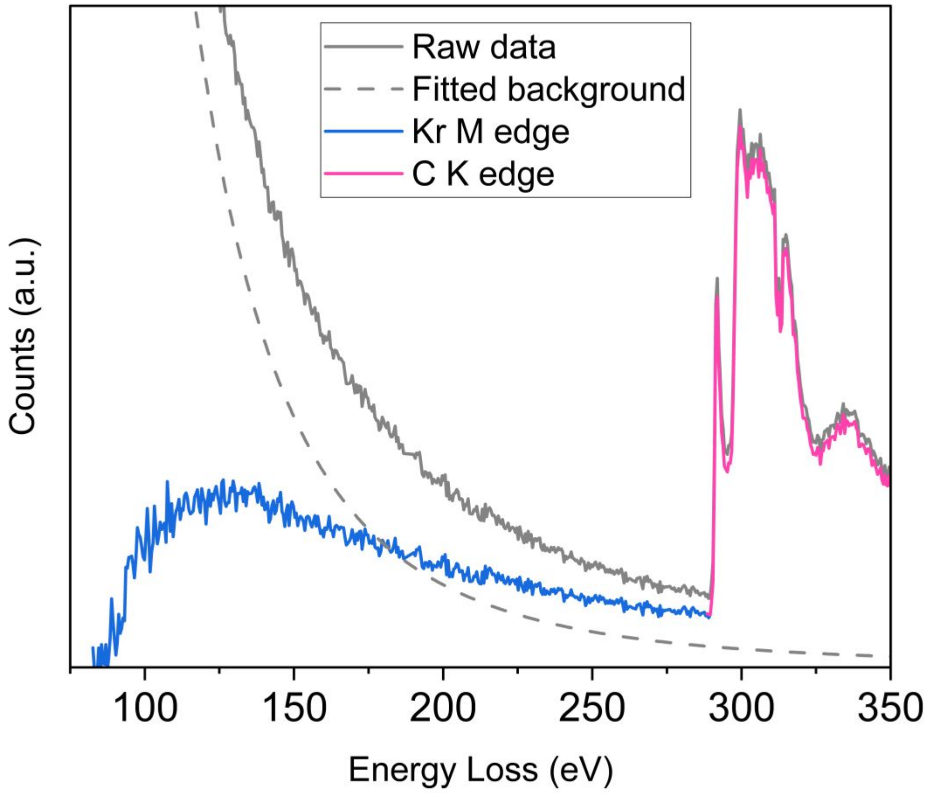

Figure S7. EEL spectrum acquired from integration of signal in green box (Figure 3h), showing raw data and the fitted background model.

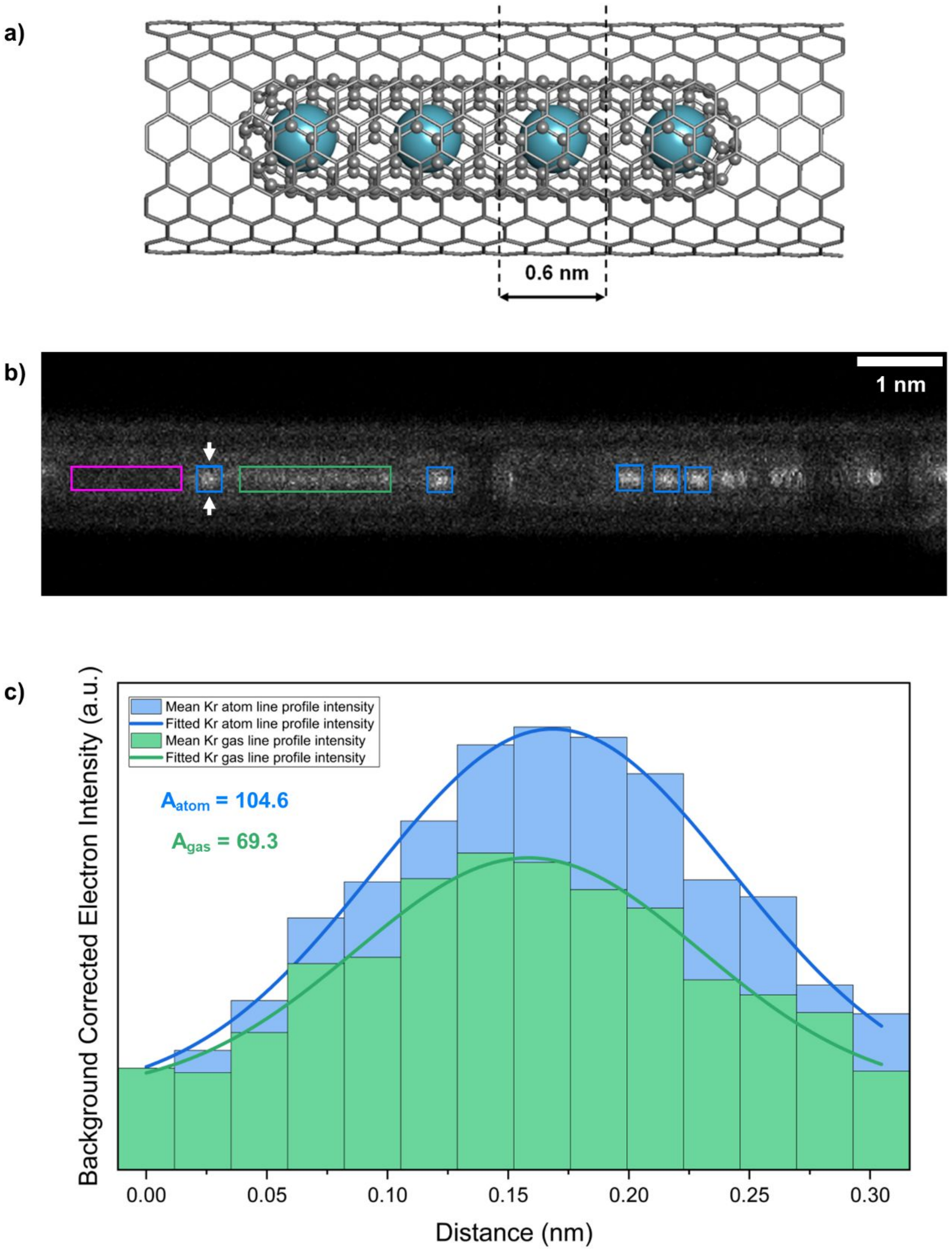

Figure S8. Measurement of relative HAADF-STEM brightness of pinned Kr atoms and mobile 1D Kr gas.

a) Calculation of space-filling of nKr@C_{60n} structures.

Length of (5,5) SWCNT containing 60 carbon atoms = 0.6 nm

Therefore 1 Kr atom per 0.6 nm on average

van der Waals diameter of Kr ~ 0.4 nm

Hence 0.4/0.6 = 2/3 of length of C_{60n} contains Kr, 0.2/0.6 = 1/3 contains empty space

b) Experimental HAADF-STEM image of an area of ($nKr@C_{60n}$)@SWCNT containing several Kr atoms pinned by the host nested nanotube (in blue boxes) and a section of 1D Kr gas (green box). The total intensity in 0.3 x 0.3 nm area (line profile direction indicated by arrows in b) was averaged over five Kr atoms (blue boxes) following background subtraction from an area of empty nested nanotube (magenta box). Similarly, the intensity in a 0.3 x 1.8 nm area (green box) was averaged over the four mobile Kr atoms forming a 1D gas.

c) Line profile analysis of stationary Kr atoms and 1D Kr gas. The mean per atom intensity of stationary Kr atoms (blue boxes in b) is shown in the blue histogram, with an area of 104.6 a.u. under the fitted Gaussian curve. The mean per atom intensity of the mobile Kr atoms (green box in b) is shown in the green histogram, with an area of 69.3 a.u. under the fitted Gaussian curve.

The relative brightness is therefore:

$$I = \frac{69.3}{104.6} = 0.663$$

This value is close to the expected average Kr occupancy of ⅔ calculated in a).

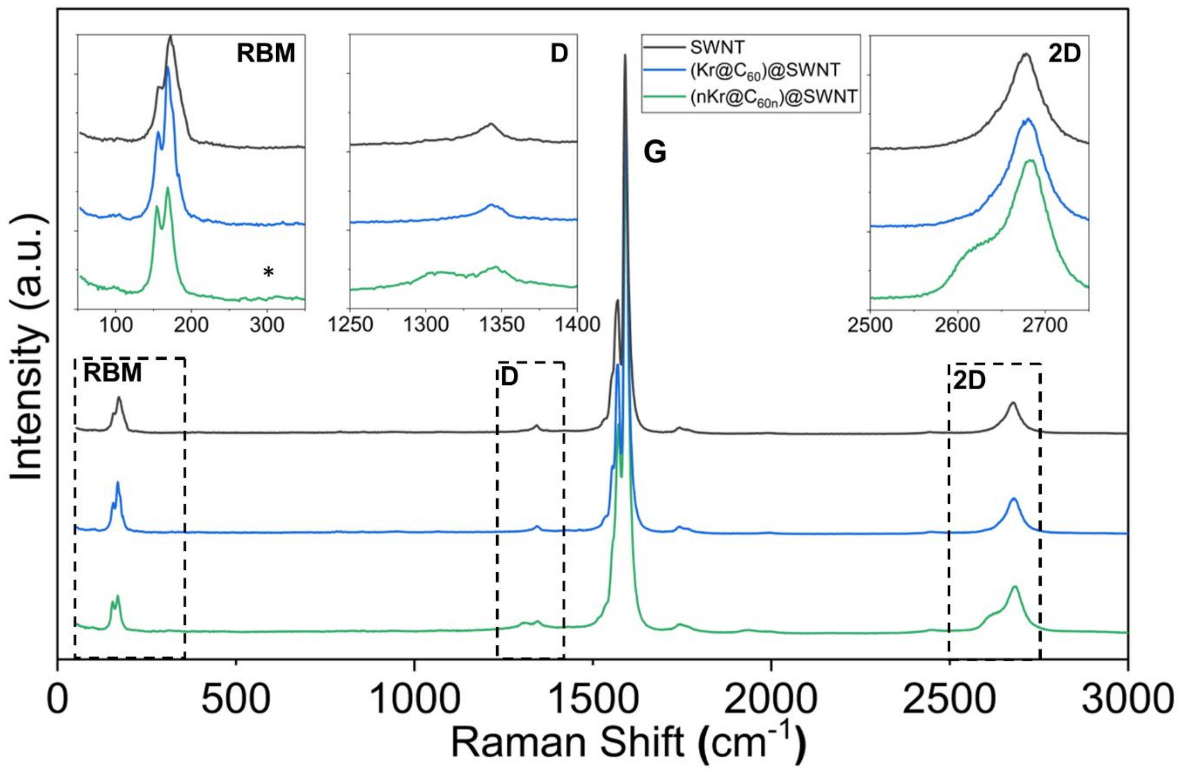

Figure S9. 532 nm resonance Raman spectra of empty semiconducting SWCNT (blue), $(Kr@C_{60})$@SWCNT (red) and $(nKr@C_{60n})$@SWCNT (grey). A 3.5 cm^{-1} red shift in the position of the principle RBM following $Kr@C_{60}$ encapsulation was observed, suggesting expansion of the nanotube to maximise van der Waals interactions with guest $Kr@C_{60}$ molecules. New D and 2D bands, and the appearance of a new RBM at ~315 cm^{-1} in polymerised $(nKr@C_{60n})$@SWCNT corresponds to new nested nanotubes of diameter ~0.77 nm, commensurate with TEM imaging.

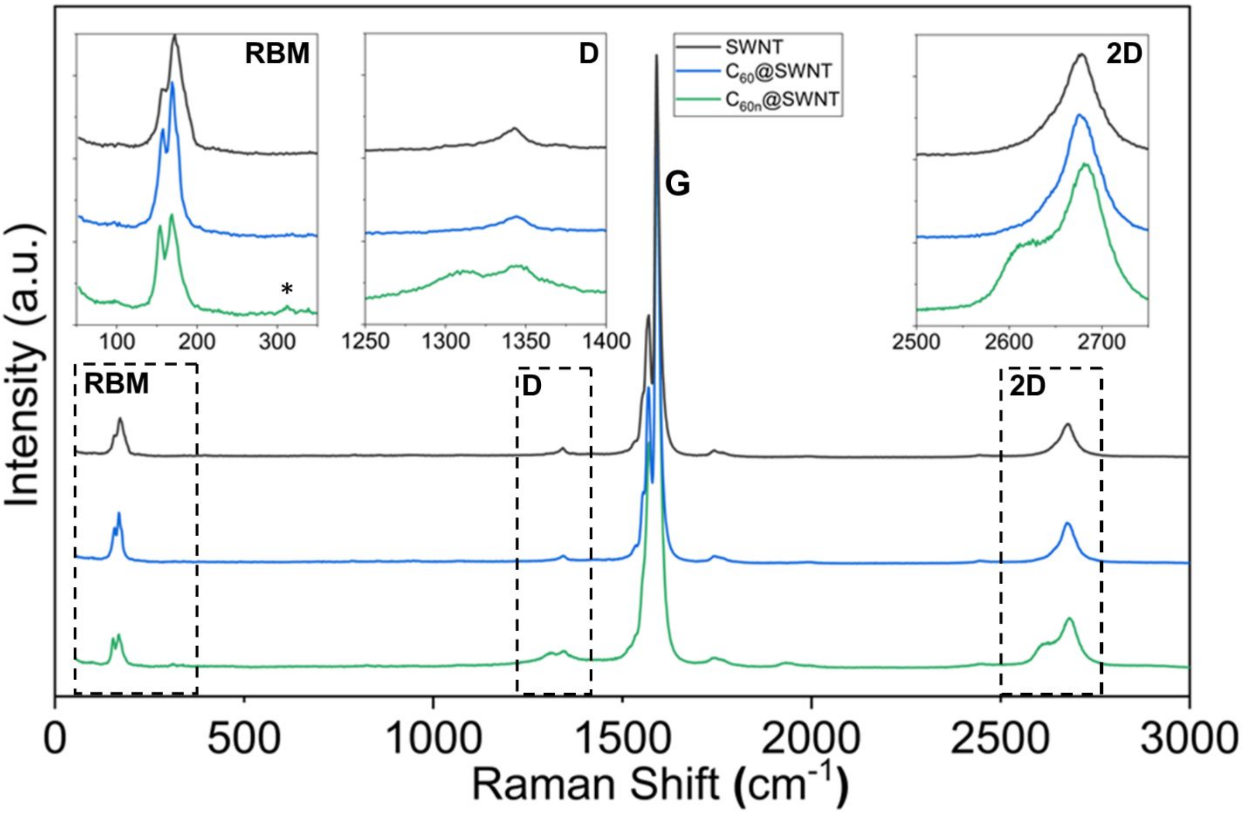

Figure S10. 532 nm resonance Raman spectra of empty semiconducting SWCNT (blue), C_{60}@SWCNT (red) and C_{60n}@SWCNT (grey). The spectra are complementary to those for SWCNT, (Kr@C_{60})@SWCNT and (nKr@C_{60n})@SWCNT shown in Figure S6, and indicate similar structural modifications.

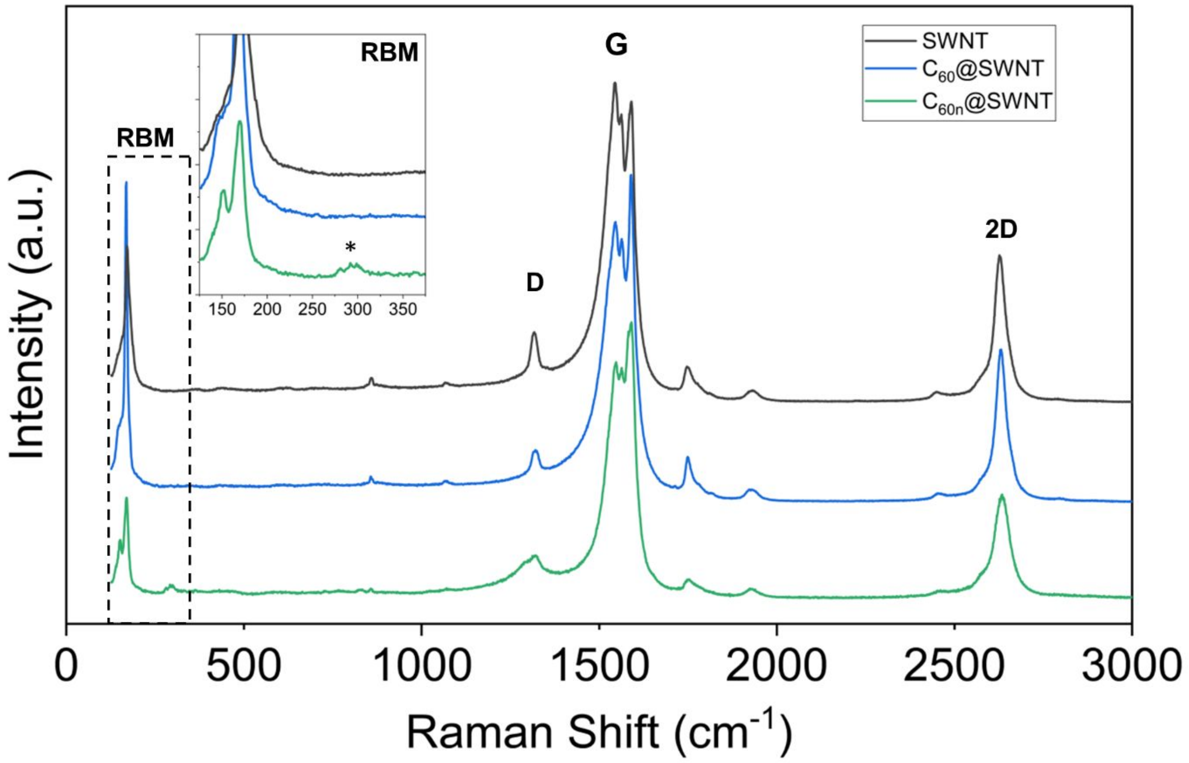

Figure S11. 660 nm resonance Raman spectra of empty metallic SWCNT (blue), C_{60}@SWCNT (red) and C_{60n}@SWCNT (grey). Observations are consistent with those seen in the equivalent (Kr@C_{60})@SWCNT samples (Figure 4).

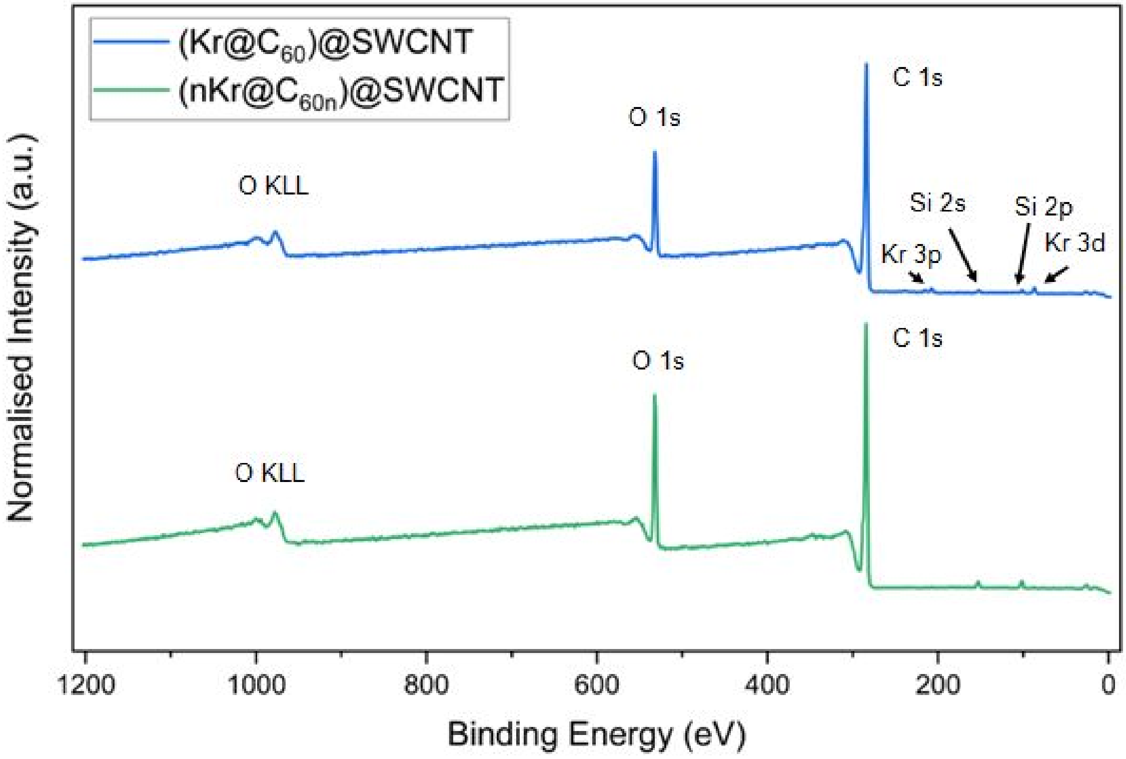

Figure S12. Wide scan XPS spectra of $(Kr@C_{60})@SWCNT$ (blue) and $(nKr@C_{60n})@SWCNT$ (green).

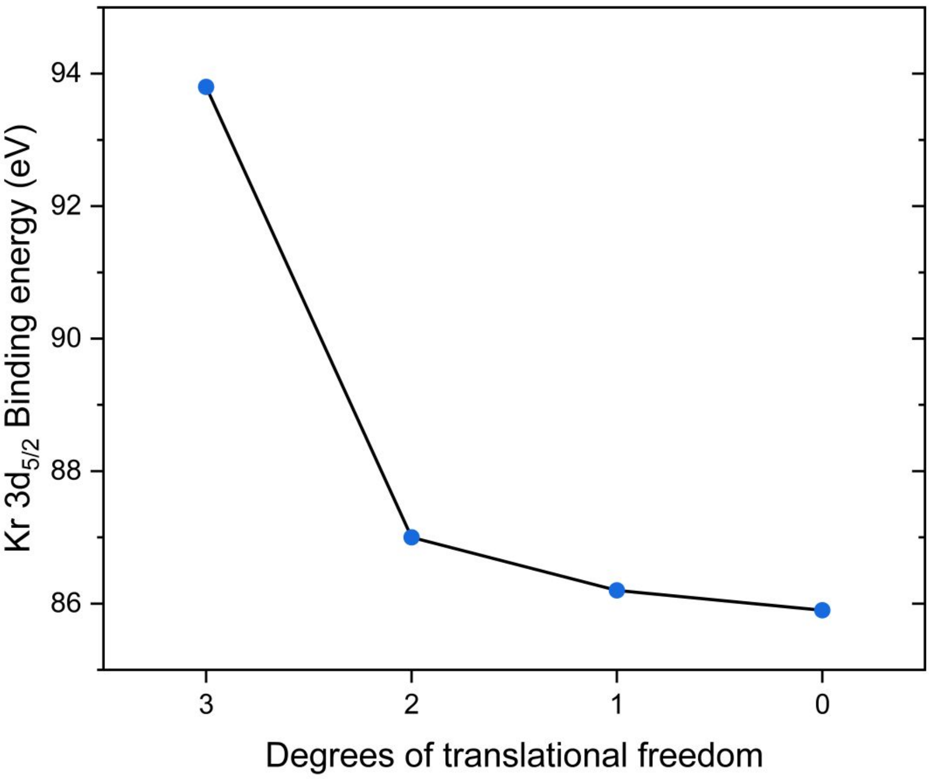

Figure S13. Plot of Kr $3d_{5/2}$ XPS peak positions with decreasing degrees of translational freedom of Kr (see Table S1)

Table S1. Kr $3d_{5/2}$ binding energies in XPS in samples where Kr has different degrees of translational freedom.

Sample	Degrees of freedom of Kr	Kr $3d_{5/2}$ binding energy (eV)	Reference
Kr gas	3	93.8	S1
Kr graphite intercalate	2	87	S2
(nKr@C_{60n})@SWCNT	1	86.2	This work
(Kr@C_{60})@SWCNT	0	85.9	This work

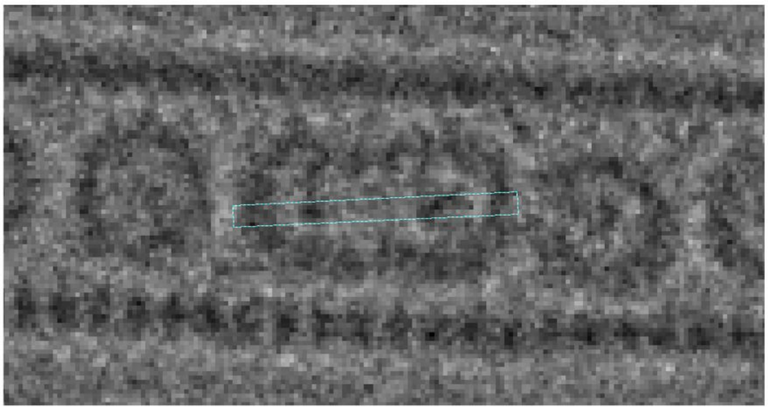

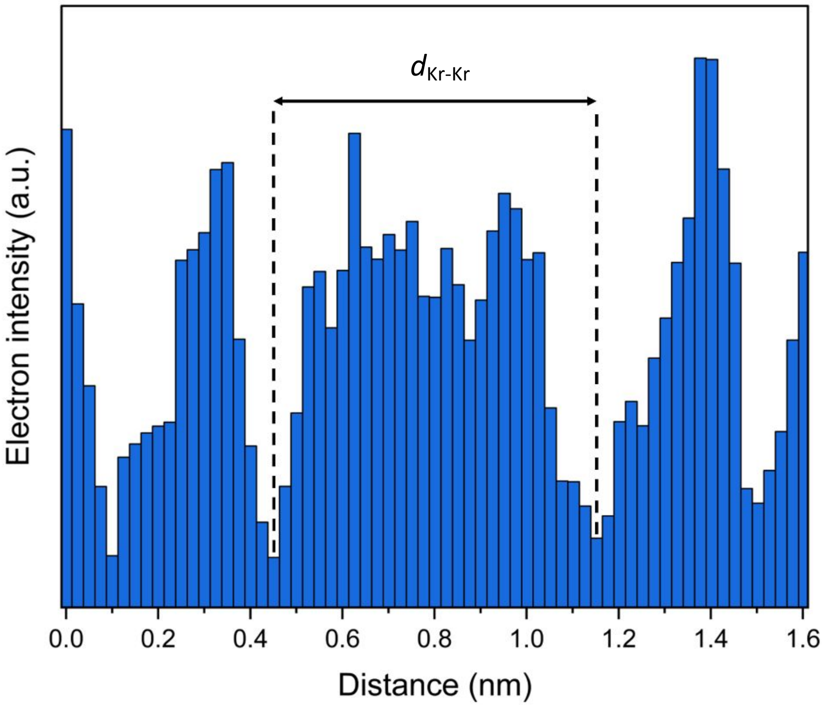

Figure S14. Methodology for the measurement of $d_{Kr\text{-}Kr}$ via line profiling. a) Representative AC-TEM image of 2Kr@C_{120}. A line profile of width = 5 px is drawn in Gatan Digital Micrograph 3.5 software, containing both areas of high contrast corresponding to Kr atomic positions during image acquisition. b) Plotted electron intensity along the line profile. Troughs correspond to dark areas. Measurement of distance between troughs corresponding to Kr positions yields $d_{Kr\text{-}Kr}$. Error is the pixel size for AC-HRTEM imaging at ±0.025 nm

Table S2. Statistical data relating to the number of surveyed areas and molecules, and the number of observed Kr@C_{60} and Kr-Kr dimerisation events.

	**Frequency**
**Time-resolved TEM datasets**	15
**Kr@C_{60} molecules observed**	296
**Discreet 2Kr@C_{120} species formed**	61
**Kr atoms pairs with $d_{Kr\text{-}Kr} \leq 0.40$ nm**	17

Justification for adaptation of Osawa-Tomanek mechanism of C_{60} coalescence to Kr@C_{60}

It is considered that the presence of endohedral Kr does not affect the mechanism of electron-beam induced fullerene coalescence when compared to C_{60}. In this context, it is noted that Kr is chemically unreactive, only forming stable or transient bonds with itself (or F, O or N), and typically behaves as a hard sphere in a gaseous state.[S3] For the case of endohedral Kr@C_{60}, the curvature of C_{60} leads to a deficiency in electron density within the internal cavity, hence it is highly unfavourable to form a covalent bond between a guest Kr atom and the concave internal surface. Accordingly, fullerene cages are considered inert hosts that coalesce under controlled electron beam irradiation to release guest Kr atoms into one continuous nano-scale capsule. At 80 kV, electron beam induced displacement of Kr from the cage is also considered highly unlikely (Equation S1).

Supporting Calculations

Maximum transferred energy to Kr atom under 80 keV electron beam:

$$T_{max}(Kr) = \frac{2M_{Kr}E(E + 2m_ec^2)}{(M_{Kr} + m_e)^2c^2 + 2M_{Kr}E}$$

(1)

Where T_{max}(Kr) is the maximum amount of kinetic energy that can be transferred to a Kr atom, M_{Kr} is the mass of a Kr atom, E is the energy of the electron beam, m_e is the mass of an electron, c is the speed of light.

$$T_{max}(Kr) = \frac{2 \times 83.798\,Da \times 1.661 \times 10^{-27}\,kg \times 80000\,eV \times 1.602 \times 10^{-19}C(80000\,eV \times 1.602 \times 10^{-19}C + 2 \times 9.109 \times 10^{-31}\,kg \times (2.998 \times 10^{8}\,ms^{-1})^2)}{(83.798\,Da \times 1.661 \times 10^{-27}\,kg + 9.109 \times 10^{-31}\,kg)^2 \times (2.998 \times 10^{8}\,ms^{-1})^2 + 2 \times 83.798\,Da \times 1.661 \times 10^{-27}\,kg \times 80000\,eV \times 1.602 \times 10^{-19}C}$$

$$T_{max}(Kr) = 3.6176 \times 10^{-19}J$$

$$T_{max}(Kr) = 2.26\,eV$$

Theoretical thermal velocity of Kr in 1D at 25 °C:

$$v = \sqrt{\frac{k_BT}{m}}$$

(2)

Where v is the 1D thermal velocity, k_B is the Boltzmann constant, T is the temperature, and m is the mass of Kr.

$$v = \sqrt{\frac{1.381 \times 10^{-23}\, m^2\, kg\, s^{-2}\, K^{-1} \times 298\, K}{83.798\, Da \times 1.661\ \times 10^{-27}\, kg}}$$

$$v = 172\, ms^{-1}$$

Approximation of average nearest-neighbour atomic separation in Kr gas at standard temperature and pressure:

Volume containing a single Kr atom:

$$V = \frac{m}{\rho}$$

(3)

Where V is volume, m is the mass of one atom, ρ is the density of Kr gas at standard temperature and pressure.

$$V = \frac{83.798\, Da \times 1.661\ \times 10^{-27}\, kg}{3.74\, kg\, m^{-3}}$$

$$V = 3.72 \times 10^{-26}\, m^3$$

Assuming each Kr atom occupies a cubic volume of 3.72 x10^{-26} m^3:

$$V = l_{cube}^3$$

$$l_{cube} = \sqrt[3]{V}$$

$$l_{cube} = \sqrt[3]{3.72 \times 10^{-26}\, m^3}$$

$$l_{cube} = 3.34 \times 10^{-9}\, m = 3.34\, nm$$

Hence the average nearest-neighbour separation between gaseous Kr atoms is ~ 3 nm.

Mean free path of free Kr gas at standard temperature and pressure:

$$\lambda = \frac{RT}{\sqrt{2}\pi d^2 N_A P}$$

(4)

Where λ is the mean free path, R is the gas constant, T is the temperature, d is the molecular diameter, N_A is Avogadro's number, and P is the gas pressure.

$$\lambda = \frac{8.3145\, J\, mol^{-1}\, K^{-1} \times 298\, K}{\sqrt{2}\pi \times (0.4\, nm)^2 \times 6.02214 \times 10^{23}\, mol^{-1} \times 1.01 \times 10^5\, Pa}$$

$$\lambda = 57\, nm$$

Approximation of 1D Kr gas pressure:

$$P = \frac{k_b T}{XA}$$

(5)

Where k_b is the Boltzmann constant, T is the temperature, A is the end cap cross-sectional area and X is the average length of nanotube available to a terminal Kr atom:

$$X = \frac{L}{N} - D$$

Where L is the available length of a nested nanotube, N is the number of Kr atoms in the 1D gas, and D is the atomic diameter of Kr.

$$X = \frac{N \times 0.6\,nm}{N} - 0.4\,nm$$

$$X = 0.2\,nm$$

Hence:

$$P = \frac{1.3806 \times 10^{-23} J\,K^{-1} \times 298\,K}{0.2\,nm\ \times (\pi \times (0.2\,nm)^2)}$$

$$P = 1.64 \times 10^{-19} J\,nm^{-3} = 164\,MPa$$

Supporting References

S1. Lundwall, M. *et al.* Preferential Site Occupancy Observed in Coexpanded Argon-Krypton Clusters. *Phys. Rev. A.* **74**, 043206-1-043206-7 (2006)

S2. Moulder, J. F.; Stickle, W. F.; Sobol, P. E.; Bomben, K. D. *Handbook of X-ray Photoelectron Spectroscopy* (Perkin Elmer Corporation, Eden Prairie, 1992)

S3. Lehmann, J. F.; Mercier, H. P. A.; Schrobilgen, G. J. The Chemistry of Krypton. *Coord. Chem. Rev.* 233-234, 1-39 (2002)
